# The effect of conflict-related violence intensity and alcohol use on mental health: The case of Colombia

**DOI:** 10.1016/j.ssmph.2024.101626

**Published:** 2024-02-08

**Authors:** Andrea Salas-Ortiz, Rodrigo Moreno-Serra, Noemi Kreif, Marc Suhrcke, German Casas

**Affiliations:** a Centre for Health Economics, University of York. United Kingdom; b Luxembourg Institute of Socioeconomic Research. Luxembourg; c Universidad de Los Andes, School of Medicine and Santa Fe University Hospital. Bogotá, Colombia

**Keywords:** Difference-in-Differences, IV, Mediation analysis, Mental health, Conflict violence, Alcohol consumption, Colombia

## Abstract

We investigated the causal impact of conflict-related violence on individual mental health and its potential pathways in Colombia. Using data from before and after the 2016 peace accord between the Colombian government and the Revolutionary Armed Forces of Colombia (FARC), we adopted a difference-in-differences empirical design combined with instrumental variables estimation. We also used formal mediation analysis to investigate a possible mediating role of alcohol consumption in the relationship between conflict exposure and mental health. Our results did not support the hypothesis that changes in exposure to conflict violence after the peace accord causally led to any changes in individual mental health. We were unable to identify a statistically significant mediating effect of alcohol consumption in the relationship between exposure to conflict violence and mental health.

## Highlights

• Two years after the 2016-peace accord, there was an overall trend of poor mental health among Meta residents.
• However, we did not find evidence about people exposed to conflict-related violence in Colombia having worse mental health.
• The lagged effect of violence on trauma is a potential reason for this.
• No evidence was found to support the self-medication hypothesis.

## 1 Introduction

Several studies have examined the relationship between conflict and physical health (Kreif, Mirelman, Suhrcke, Buitrago, & Moreno-Serra, 2022; Lutz & Mazzarino, 2019), finding - overall - that being exposed to conflict violence harms people's physical health. However, robust evidence about the impact of conflict-related violence on mental health (and its potential pathways such as those via trauma or daily stressors) is comparatively scarce, primarily due to the challenges involved in disentangling causal relationships in this context. Exposure to violence may not be exogenous: people who are exposed to different levels of conflict violence may have different experiences of mental health, for reasons other than the conflict, and there might also be reverse causality (Bratti, Mendola, & Miranda, 2015; Do & Iyer, 2009). The latter situation might arise if, for instance, people with mental health disorders are systematically more prone - or less able - to leave high-conflict localities.

Most of the literature on the impact of war or conflict-related violence on individual-level mental health has paid little attention to these issues. In this paper, we exploited features of the Colombian conflict and peace process to investigate the causal relationship between conflict violence and mental health. In December 2016, the Colombian government, and the Revolutionary Armed Forces of Colombia (FARC) signed a peace agreement^1^ after three years of negotiations and after at least four failed peace talks.^2^ After six decades of civil conflict, mental health disorders related to violence exposure seem to have become highly prevalent in Colombia. Based on data from the most recent National Mental Health Survey (2015), over 40% of adults report exposure to a traumatic event (Ministerio de Salud y Protección Social, 2015). Colombian studies have identified anxiety as the most prevalent mental health disorder, followed by alcohol abuse (de la Espriella Guerrero et al., 2016, pp. 76–88; Rincon-Hoyos, Castillo, & Prada, 2016). Mental health disorders impose a significant economic burden on the country (Tamayo, Simbaqueba, Palomino, & Simbaqueba, 2016).

We took advantage of the variation in the level of conflict-related violence induced by the 2016 peace agreement across Meta's municipalities^3^ to

Estimate the causal effect of conflict violence on mental health, using data from before and after the 2016 peace agreement. Given the lack of randomisation of conflict-related violence across individuals, there might be factors confounding the relationship between violence exposure and mental health. This includes potentially unobserved factors affecting mental health that are correlated with conflict-related violent events (omitted variables), and reverse causality. If these sources of bias are not addressed, estimates of the causal relationship between conflict violence and mental health may simply reflect a spurious correlation. In this paper, we combined two econometric approaches to isolate the causal effect of conflict violence exposure on mental health: difference-in-differences (DiD) and instrumental variable (IV) estimation. The DiD approach entails comparing changes in individuals’ mental health status over time between people highly exposed to conflict-related violence and individuals unexposed or lightly exposed, allowing for the exposed and unexposed groups to differ in predictors of mental health, as long as these predictors do not change over time. The addition of the IV method allows us to further control for potentially unobserved factors that influence both mental health and violence, and that change over time. As our instrumental variable - an exogenous factor that affects conflict violence, but does not directly affect mental health - we selected coca production in the municipality of the individual, drawing upon insights into the historical dynamics of conflict-related violence, and the abundant evidence linking cocaine production and violent events in Colombia (Beeder, 2020; Rubio-Ramos, 2023).

Another relevant aspect in the exploration of the impact of conflict violence on mental health is understanding the mechanisms through which this effect may take place. One is the *self-medication* hypothesis (Miller, Kulkarni, & Kushner, 2006; Miller & Rasmussen, 2010), which states that people exposed to conflict-related violence may abuse alcohol consumption as a coping mechanism, which – in turn – is likely to impact their mental health. While several studies have explored this hypothesis in low-and middle-income countries (Branas et al., 2013; Lo, Patel, Shultz, Ezard, & Roberts, 2017; Ramachandran et al., 2019; Roberts et al., 2014), some studies have explored the relationships between exposure to conflict violence, the presence of mental health disorders, and alcohol consumption in Colombia (de la Espriella Guerrero et al., 2016, pp. 76–88; Gómez-Restrepo et al., 2016; Londoño, Romero, & Casas, 2012; Marroquín Rivera, Rincón Rodríguez, Padilla-Muñoz, & Gómez-Restrepo, 2020; Ramírez et al., 2014; Roberts & Browne, 2011; Tamayo Martínez et al., 2016). However, all of these studies are descriptive and do not shed light on the causal mechanisms at play. Therefore, capitalising on our data and econometric strategy, we also explored the role of harmful alcohol consumption as a potential mechanism through which conflict-related violence might impact mental health.

Our focus on the case of the Meta departamento in Colombia is motivated by the fact that the FARC were the dominant guerrilla group in that departamento since the 1970s, and Meta has been one of the Colombian departamentos most affected by conflict violence. Meta ranks among the top five regarding the incidence of conflict-related violence acts, comprising terrorist attacks, war actions, attacks on populations, selective murders, kidnappings, child recruitment, massacres, enforced disappearance, damage to properties, and sexual violence. Meta accounted for 5.5% of the total number of victims of conflict-related violence between 2000 and 2021, only topped by two other departamentos: Antioquia and Norte de Santander (Centro Nacional de Memoria Histórica, 2022).

Our work contributes to filling two gaps in the literature. First, despite Colombia being one of the countries that have been most affected by long-standing conflict-related violence, most of the existing evidence about mental health consequences is descriptive and focused on correlations, as concluded by recent systematic reviews (Peevey, Flores, & Seguin, 2022; Tamayo Martínez et al., 2016). Second, most of the quantitative studies in the international literature have focused on the relationships between conflict violence and physical health, whilst rigorous causal assessments of the impact of conflict on mental health and related risk factors are missing. Studying the mental health impacts of conflict violence is relevant not only from a Colombia-specific perspective, but also at a global level. We adopt a rigorous causal approach to tackle different sources of bias in the estimated relationship between conflict violence exposure and mental health, using the Colombian peace process as a natural experiment. Beyond looking at the total effect of conflict on mental health, we also investigate potential mechanisms behind this impact. The remainder of the paper is organised as follows. Section 2 describes the methods and the econometric strategy used. Section 3 provides detailed information about the data and key variables. Section 4 presents the main results and additional analyses, whilst the final section discusses the results and concludes.

## 2 Methods

### 2.1 Conceptual framework for the estimation of causal effects

**Fig. 1:**
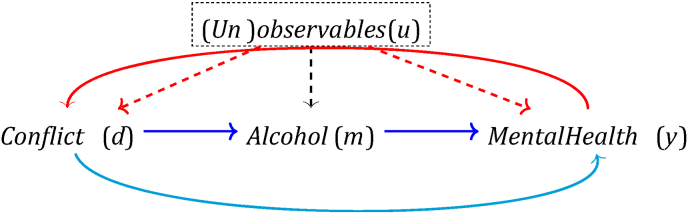
Potential endogeneity in the estimation of the causal effect of conflict violence on mental health.

Protracted conflicts – i.e. long-running confrontations – are likely to put mental health at risk (Steel et al., 2009), which may happen in various ways: conflict can have direct effects, by increasing stress levels (Hobfoll et al., 1991), and/or indirect effects, by affecting certain behaviours, such as harmful substance use (e.g. self-medication). Analysing either the total effect of conflict violence on mental health and separating this effect from its direct effect (light blue line in Fig. 1) and the indirect effect mediated by alcohol use (second dark blue line) requires addressing potential confounding factors and reverse causality.

Confounding factors (*u*) of the causal effect of conflict on mental health are variables that affect both conflict violence and also have an independent effect on mental health. When the indirect effect of conflict violence through alcohol use is of interest, it is also important to consider variables that affect both alcohol consumption and mental health. In either case, we consider individual demographic characteristics, such as age and sex, and social and economic factors, including marital status, level of education and job type. The role of these factors has been suggested by a previous study (León-Giraldo et al., 2021, León-Giraldo et al., 2021, León-Giraldo et al., 2021), the findings of which indicated that socioeconomically disadvantaged individuals - in terms of employment status and education levels - were at a higher risk of developing mental health disorders. We might also expect other external confounding elements at the macro level, such as social, political, or economic changes. The second challenge – reverse causality between conflict and mental health (depicted by the red line) – can arise if individuals with mental health disorders cause conflict violence.

We addressed these challenges by using a combination of our rich dataset and a natural experiment setting in Colombia. The DiD approach combined with instrumental variables, described in detail below, accounts for observed confounders (demographic and socioeconomic factors), unobserved individual-level confounders that change and do not change over time, and for common time trends.

### 2.2 Strategy to identify causal effects

#### 2.2.1 Definition of highly and lightly or unexposed groups

The identification of causal effects relies on the fact that the Colombian 2016 peace accord exposed individuals to a heterogeneous change in conflict-related violence across municipalities. Although the 2016 peace accord applied to all departamentos and municipalities at the same time, the expected reduction of conflict intensity induced by the agreement varied according to the level of conflict violence observed in each municipality before the peace accord. Municipalities that were highly affected by conflict violence before the accord can be expected to have seen larger reductions in violence post-accord, compared to municipalities that were lightly affected or unaffected by pre-accord conflict violence. Therefore, the peace accord should have induced a *differential change* in exposure to conflict violence between individuals living in highly affected municipalities and those living in lightly affected or unaffected municipalities. These different changes in levels of exposure to conflict violence can be exploited as a natural experiment, within a standard DiD estimation framework.

**Fig. 2:**
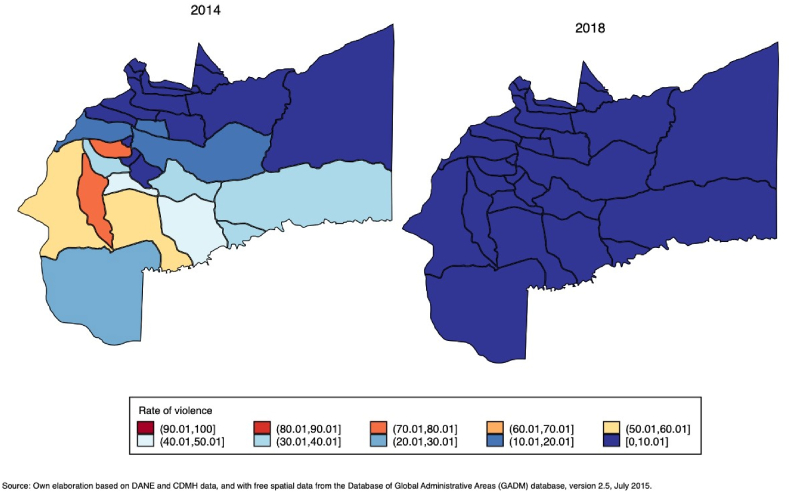
Number of conflict-related violent events per 100,000 inhabitants in 2014 and 2018.

Figure (2) depicts the level of conflict-related violence across municipalities in 2014, which decreased almost to zero in 2018. Additionally, Figures (A1) and (A.2), in the Electronic Supplementary Material (ESM), show the evolution in the standardised^4^ number of violent events in each of the 29 municipalities of the Meta departamento from 2000 to 2018. These figures indicate that before the 2016 peace accord, there was already a decreasing trend in conflict-related violence.^5^

By classifying municipalities according to conflict intensity in 2014 (defined by the median number of conflict-related violence events), we observe that almost all of the municipalities in the highly exposed group decreased their rate of violent events to zero by 2018. By contrast, most of the municipalities in the lightly or unexposed group had smaller or already zero violence rates (see Figures (A2) and (A.3) in the ESM). This evidence supports our argument regarding the existence of differential changes in exposure to conflict violence post-peace accord.

Therefore, our main argument for causal identification is that people living in heavily affected municipalities are expected to benefit more from the cessation of hostilities compared to individuals living in lightly or entirely unaffected municipalities, where conflict violence was already much lower before the peace accord. In our study, we define exposed and lightly or unexposed groups according to the level of conflict-related intensity in the municipalities where individuals lived in 2014 (i.e. pre-peace accord).

### 2.3 Difference-in-differences approach

Our first strategy to deal with the unobserved confounding is to introduce individual fixed effects (FE) within a DiD setting. This will eliminate any bias coming from time-invariant unobserved heterogeneity that influences both conflict intensity and mental health. To this end, we model individual mental health as a function of conflict-related violence intensity in 2014, interacted with the post-agreement year (i.e. 2018), and controlling for time-varying covariates, individual-specific time-invariant effects (*θ*_*i*_), a time-specific component (*θ*_*t*_), and another term that captures the idiosyncratic time variant error (*ϵ*_*imt*_), as follows:

(1) *yimt* = *α* + *τdm,* 2014 ∗ *t*2018 + *γXimt* + *θt* + *θi* + *ϵimt*

where, *y*_*imt*_ represents a binary variable (1: mental health disorder, 0: otherwise), by individual *i* who lives in municipality *m*, measured at time *t*, and *τ* represents our parameter of interest. This coefficient represents the estimated additional change in the probability of developing mental health disorders two years after the peace accord by people highly exposed to conflict-related violence, compared to people who were not exposed or lightly exposed to conflict violence in 2014. Using Equation (1) to estimate *τ* takes into account two sources of potential endogeneity: one source resulting from time-invariant unobservable factors that are potentially correlated with (both) individual exposure to different levels of conflict violence intensity and individual mental health (captured by *θ*_*i*_), and another from aggregate shocks that affect mental health for all individuals at the same time (captured by *θ*_*t*_). Equation (1) is our two-way fixed effects model. We also estimated a different version of Equation (1), where our mediator is included:

(2) *yimt* = *α* + *τdm,* 2014 ∗ *t*2018 + *γ*1*mimt* + *γ*2*Ximt* + *θt* + *θi* + *ϵimt*

where *m*_*imt*_ represents a binary variable (1: harmful alcohol use, 0: otherwise), this model allows for the assessment of the direct effect of conflict exposure on mental health, which is not mediated through alcohol consumption. Both models require that the estimated standard errors are adjusted for arbitrary types of serial correlation and heteroscedasticity. Therefore, we use bootstrapped panel standard errors clustered at the municipality level in our estimations.

### 2.4 Instrumental variable estimation

We complement the DiD approach by estimating Equation (1) using an IV strategy, to account for further sources of endogeneity that *θ*_*i*_ and *θ*_*t*_ cannot capture. The IV strategy involves estimating Equation (1) using a two-stage least squares (2SLS) model. In selecting our instrument, we built on the literature that investigates illegal drug production as a factor influencing conflict violence (Angrist & Kugler, 2008; Mejia & Restrepo, 2013). Specifically, we exploit the fact that the FARC consolidated its economic and organisational power due to the *economy of cocaine* (Aguilera Peña, 2013; Contreras, 2018a; González, 2014). In Meta, this involved the FARC taking advantage of the rich natural resources found there to support the large-scale production of coca (cocaine's main ingredient) as a financial source of support for the guerrilla (Lavaux, 2006). Hence, our IV strategy relies on the fact that the endemic geographic characteristics of some Meta municipalities were ideal for producing coca (i.e. abundance of hydric resources, low temperature, and altitude).

In this context, we expected to observe an inverse relationship between coca production and conflict-related violence: among those municipalities where coca was intensively produced, violence was expected to be low, since these municipalities were already under the control of the FARC. By 2014, the municipalities where the FARC had established themselves as dominant, taking control of resources and coca production, were all but determined already by the decades of the long historic process of conflict. Meta (as other FARC-dominated areas) was already experiencing a process of relatively stagnant and lower levels of conflict violence than in previous decades. This was due also to various (official and unofficial) ceasefires declared to support the Havana talks between the government and the FARC, which began in 2012 and would lead eventually to the 2016 peace agreement.

Next, we argue that coca production, as an economic factor, is an exogenous predictor of municipal conflict violence intensity, as such production was mostly determined by geography and the availability of water resources (Mejia & Restrepo, 2013; Reilly & S, 2019). There are two reasons behind this. First, access to water is one of the most important inputs not only for the cultivation of coca but also to produce cocaine. Access to water sources varies across Meta municipalities, unlike other environmental factors that are also relevant for the suitability of coca production in a given area, such as altitude and temperature (IDEAM I. de H. Meteorologia y Estudios Ambientales, 2022). Second, water resources in the form of the presence of rivers have been of high relevance for the distribution of coca leaves and cocaine. Particularly, the Arari, Duda and Guayabero rivers, all located in the Cordillera Oriental and crossing several Meta municipalities, have been used as bastions by the FARC (Centro Nacional de Memoria Histórica, 2014), and have significantly influenced the capacity for cocaine production. For further evidence regarding the influence of coca and cocaine production on conflict intensity, we refer the reader to the related literature (Aguilera Peña, 2013; Beeder, 2020; Contreras, 2018b; González, 2014; Mejia & Restrepo, 2013; Reilly & S, 2019; Rubio-Ramos, 2023).

To alleviate the concern that the direction of causality may run the other way around – from conflict violence to coca production (Barrera, Gómez, & Velasco, 2023) – we noted that it was sensible to expect that conflict violence levels were not an important determinant of differences in the levels of coca production across municipalities during the 2014–2018 period, with such differences originating instead from historical, geographic, and economic conditions. We also inspected the validity of our IV strategy through several tests, such as those for relevance and strength (Kleibergen-Paap LM and Wald *F* tests), which explore whether the instrument has insufficient explanatory power to predict the endogenous variable in the model (Windmeijer, 2021). These tests relax the assumption that first-stage errors are independent and identically distributed (i.i.d). Although the exogeneity of an instrument cannot be tested formally, we investigate whether coca production was correlated with the probability of developing mental health disorders before the peace accord took place, with and without conditioning on socioeconomic and household characteristics. If there was a statistically significant correlation, then this would be indicative of the exogeneity assumption not holding.

As for other potential IVs for our study, there were several initial candidates for instrumenting conflict-related violence. These include geographical factors, such as whether the Arari, Duda, and Guayabo rivers crossed a given municipality in Meta, thus favouring coca cultivation in the municipality. Other environmental factors related to the suitability of coca production were also explored, such as altitude and temperature. These are factors closely related to conflict violence, while arguably unrelated to mental health outcomes or other potential confounding factors. However, these are either time-invariant or have very little variation over time, whereas our identification strategy requires instrumental variables that change over time.

#### 2.4.1 Testing for reverse causality

As explained previously, should there exist reverse causality running from mental health outcomes to conflict violence exposure, our IV estimates would be biased. Thus, we adopted a simple test to assess whether there is evidence in this direction, by estimating a regression of conflict violence in 2014 as the dependent variable^6^ against coca production in a given year (*z*_*m,t*_), and its lead terms (*z*_*m,t*+1_ and *z*_*m,t*+2_)), along with control variables and individual and time-fixed effects, as follows:

(5) *dm,* 2014 = *α* + *φ*1*zm,t* + *φ*2*zm,t*+1 + *φ*3*zm,t*+2 + *γXimt* + *θt* + *θi* + *ωimt*

To test the robustness of the estimates, we estimate two models, one that includes one coca production lead term *z*_*m,t*+1_, and another that includes two lead terms *z*_*m,t*+1_ and *z*_*m,t*+2_. We examine whether - in the case of using a single lead term - the *φ*_2_ coefficient in Equation (5) is statistically different from zero, or - in the case of two lead terms - whether a joint test on coefficients *φ*_2_ and *φ*_3_ indicates that such coefficients are jointly statistically different from zero. Non-rejection of the null hypothesis that the tested coefficients are equal to zero would offer reassurance that coca production is not driven by conflict violence levels during our study period.

### 2.5 Does alcohol use mediate the effect of conflict violence on mental health?

We explored the indirect effect of conflict-related violence via alcohol consumption on individual mental health, following a formal causal mediation analysis approach. In short, mediation analysis aims to assess the causal mechanisms along the pathway between a given treatment (here: conflict violence) and an outcome (here: mental health), disentangling the total effect into an indirect effect that operates via one or several observable intermediate factors (mediators) and a direct effect that reflects any impact not captured by the observed mediators. To explore the self-medication hypothesis, here we focus on one mediator, i.e. harmful alcohol consumption. In our causal conceptual framework (Fig. 1), the direct and indirect effects are depicted with the light and dark blue lines, respectively.

For this analysis, we build on the DiD-IV framework described previously and amend it using the three-equation framework proposed by Hsia, Tai, Kao, and Lin (2021) (Hsia et al., 2021). With the first equation, we estimate the total effect effect of conflict exposure on mental health. The second equation expands the previous analysis, by also controlling for changes in harmful alcohol use. The coefficient on conflict exposure in this equation can be interpreted as the natural direct effect of conflict on mental health. Finally, the natural indirect effect can be estimated by employing a third regression, that models harmful alcohol use as a function of changes in levels of exposure to conflict violence, and combining the estimated coefficients from the second and third equations. In all three equations, we control for individual- and time-specific effects, as well as for endogeneity in the violence exposure variable. The latter implies making use of two-way fixed effects and using coca production as IV. Because the causal mediation makes a further assumption - no unmeasured confounders for the mediator-outcome relationship - that is unlikely to hold in our case, we interpret the total indirect effect, and estimated natural direct and natural indirect effects as exploratory, rather than causal. The mediation analysis is formally described in the ESM section A.

### 2.6 Additional analyses and robustness checks

We investigated a further mechanism potentially driving exposure to conflict violence and mental health: the *daily stressors* hypothesis (Miller & Rasmussen, 2010). This hypothesis states that mental health may not necessarily improve – and may even deteriorate - once a conflict ends. This is because of the social and material conditions that affect people's day-to-day lives, which may have been important drivers of mental health conditions during the conflict and may remain so after conflict de-escalation. This can include changes in the family structure and functioning, labour market conditions, social marginalisation, or inadequate housing (Miller et al., 2006; Miller & Rasmussen, 2010). We undertook an exploratory analysis whereby we ran mediation analysis equations sequentially, including in the regression combinations of proxy variables for these factors in a stepwise manner. These proxy variables are the demographic and socioeconomic confounding factors mentioned previously in section 2.1. In the first model, we only include the variable of interest (conflict-related violence interacted with the post-exposure period). A second specification includes the variable of interest plus only individual age and education as controls. A third specification includes the variable of interest plus marital status, and so forth. Finally, we run a full model that includes variables of interest and all the proxy variables for daily stressors as covariates. The idea behind this analysis is that if there are statistically significant changes in the coefficient indicating changes in conflict violence in the post-exposure period when a variable (or group of variables) is added, then this latter variable would be a relevant daily stressor and hence a further mediator.

## 3 Data

We administered a tailor-made household survey called "Conflicto, Paz y Salud (CONPAS)" in the Meta departamento of Colombia in 2018. The survey sample was selected through a probabilistic multistage sampling. The survey is representative of the urban and rural populations of the Meta departamento. Retrospective data for year 2014 and information from 2018 were collected from the same respondents. 1309 households were interviewed in total. Respondents were adults aged between 18 and 64 years of age. A specialised survey company was hired to help respondents recall events, situations and feelings that took place in 2014. Further detailed information on the survey is available elsewhere (León-Giraldo et al., 2021, León-Giraldo et al., 2021, León-Giraldo et al., 2021; León-Giraldo et al., 2021, León-Giraldo et al., 2021, León-Giraldo et al., 2021; León-Giraldo et al., 2021, León-Giraldo et al., 2021, León-Giraldo et al., 2021).

We proxied exposure to conflict-related violence by the total number of conflict-related violent events per 100,000 inhabitants observed in the individual's municipality of residence *m* (*m* = 1*, …,*29) in 2014 (i.e. pre-2016 peace accord). The total number of conflict-related events is the sum of events comprising terrorist attacks, war actions, attacks on populations, selective murders, kidnappings, child recruitment, massacres, forced disappearance, damage to property, any kind of sexual violence, and landmine explosions that took place in 2014 in a given municipality. We used this information to construct a binary variable indicating the intensity of conflict-related violence to which individuals were exposed before the peace accord. This conflict intensity indicator takes the value of 1 if the individual lived in a municipality with a high intensity of violence, defined as a total number of conflict-related events above the sample median value in 2014, and 0 otherwise. Data on conflict-related violent events came from the National Centre for Historical Memory of Colombia (CNMH).

Individual mental health status was measured using the Self-Reporting Questionnaire-25 (SRQ-25), developed by the World Health Organisation (WHO). This instrument assesses the risk of an individual developing mental health disorders. The WHO guideline to construct this variable states that if a person answers “yes” to eight or more questions, then this person presents a positive tendency to develop mental health disorders (Beusenberg, Orley, & Health W. H. O. D. of M, 1994). Harmful alcohol consumption was measured at the individual level via the Alcohol Use Disorders Identification Test (AUDIT), also developed by WHO. This instrument has 10 questions, with responses scored with values of 0, 1, 2, 3 or 4. When adding up responses across the ten questions, scores can range from 0 to 40, where 0 indicates that the person has never had any problems with alcohol. Scores from 1 to 7 suggest low-risk consumption, while scores above 8 suggest harmful alcohol consumption (Babor, Higgins-Biddle, Saunders, & Monteiro, 2001, p. 41). In our analysis, these two variables were binary and took the value of 1 to indicate a tendency to develop mental health disorders or harmful alcohol consumption and 0 otherwise.

CONPAS collected rich information about other individual characteristics. This included individual-level data on sex (1 for men and 0 otherwise), ethnicity, which is a categorical variable.

(white, indigenous, mestizo, and others,^7^ age was categorised into three groups: young adults (those younger than 35 years of age); middle-aged adults (between 36 and 65 years of age) and older adults (older than 66 years of age) (Franssen, Stijnen, Hamers, & Schneider, 2020). We also include information on marital status, categorised into three groups (single, married, and other, including separated, divorced or widowed). Information on formal education comprises the following categories: no education, up to secondary school, and above secondary school, for example, technical school, higher or postgraduate degrees. Occupation was categorised as: in paid job (e.g., employee of a private company or the government; day labourer; domestic employee; self-employment, farm worker); non-work paid (e.g., pensioners); unpaid job (e.g., family carer without remuneration) and unemployed (e.g., students, individual working in household duties or being permanently incapacitated to work).

At the household level, the CONPAS data included information on assets: whether the household had: electricity; running water; refrigerator; internet services; television; telephone; computer; laundry machine; car; bicycle; radio; toilet connected to sewer, and concrete flooring. With this information, we proxied household wealth by calculating an index for each individual using principal component analysis following (O’Donnell, Van Doorslaer, Wagstaff, & Lindelow, 2007).^8^ From the distribution of these indices, we created a categorical variable indicating the quartile of the household index distribution to which the individual belongs, with higher quartiles indicating higher levels of household wealth. Finally, CONPAS also collected data on monthly household expenditure, encompassing utilities, rent, food, toiletries, and clothes. We expressed this expenditure in real terms and 2018-US dollars^9^ and used it as a continuous variable in our analyses.

## 4 Results

### 4.1 Descriptives

Table (1) describes the extent to which conflict exposure groups were different in observable characteristics, before and after the peace accord. We present the mean value, the difference in means, and a *p*-value associated with a mean-comparison test^10^. Overall, the proportion of people with a tendency to develop mental health disorders increased over time, while the share of people with harmful alcohol consumption decreased. Coca production in the municipality of residence also decreased over time for both groups.

Before the accord, the proportion of people at risk of developing mental disorders (SRQ+) was greater among those highly exposed to violence (20%) than among those lightly or unexposed (17%). In the post-accord period, these proportions increased to 38% and 36% in both groups, although differences in proportions across groups were not statistically significant in either period. The proportion of people with harmful alcohol use (AUDIT+) was slightly higher in the highly exposed (16%) compared to the lightly exposed group (15%) at baseline. Post-accord, these proportions decreased in both groups, to 11% and 12%, respectively. Differences in these proportions across groups were not statistically significant in either period. Municipalities with higher exposure to violence had a much higher production of coca in both periods, although this production decreased in both groups of municipalities after the peace accord. As for other selected variables, Table 1 shows that in those municipalities highly affected by conflict violence, there were higher proportions of men and lower shares of individuals aged below 35, single, or with formal education above secondary school. Moreover, individuals in areas highly exposed to violence tended to belong to households with lower expenditure and to the lowest (poorest) quartile of our asset index.

**Table 1:** Average characteristics of individuals pre- and post-peace accord.

	Pre-accord (2014)				Post-accord (2018)			
	d = 0	d = 1	Diff.	p-val	d = 0	d = 1	Diff.	p-val
Outcome								
SRQ+	0.17	0.20	−0.03	0.12	0.36	0.38	−0.01	0.60
*Mediator*								
AUDIT+	0.15	0.16	−0.01	0.50	0.11	0.12	−0.01	0.71
**Covariates**								
Men	0.42	0.51	−0.10	0.00	0.42	0.51	−0.10	0.00
***Age***								
Below 35 years old	0.42	0.32	0.10	0.00	0.34	0.24	0.10	0.00
36–65 years old	0.49	0.59	−0.09	0.00	0.52	0.60	−0.09	0.00
Above 66 years old	0.09	0.09	−0.00	0.86	0.14	0.15	−0.01	0.67
***Ethnicity***								
White	0.34	0.38	−0.04	0.10	0.34	0.38	−0.04	0.10
Indigenous	0.03	0.07	−0.03	0.01	0.03	0.07	−0.03	0.01
Mestizo	0.50	0.34	0.16	0.00	0.50	0.34	0.16	0.00
Other	0.13	0.22	−0.09	0.00	0.13	0.22	−0.09	0.00
***Marital Status***								
Single	0.15	0.10	0.05	0.00	0.09	0.06	0.03	0.02
Married	0.63	0.67	−0.03	0.20	0.62	0.62	−0.00	0.96
Other Status	0.21	0.23	−0.02	0.40	0.29	0.32	−0.03	0.20
***Education***								
None or below sec.	0.79	0.90	−0.11	0.00	0.74	0.88	−0.14	0.00
Above secondary school	0.21	0.10	0.11	0.00	0.26	0.12	0.14	0.00
***Job Status***								
Paid job	0.66	0.68	−0.01	0.60	0.58	0.63	−0.05	0.07
No-work paid	0.03	0.02	0.01	0.33	0.04	0.02	0.02	0.04
Unpaid job	0.30	0.29	0.00	0.87	0.37	0.34	0.03	0.35
Unknown job	0.01	0.01	0.00	0.83	0.01	0.01	0.00	0.56
***Household circumstances***								
Average expenditure+	307.55	245.51	62.03	0.00	344.34	262.96	81.38	0.00
Asset Index:q1	0.11	0.43	−0.33	0.00	0.11	0.43	−0.32	0.00
Asset Index:q2	0.23	0.28	−0.06	0.02	0.23	0.28	−0.06	0.02
Asset Index:q3	0.29	0.19	0.10	0.00	0.28	0.21	0.08	0.00
Asset Index:q4	0.37	0.09	0.28	0.00	0.38	0.08	0.30	0.00
***Municipality characteristics***								
Coca production (Hectares)	0.07	768.29	−768.22	0.00	0.00	483.53	−483.53	0.00
Observations	1309				1309			

Notes: d = 0: individuals in lightly affected municipalities. d = 1: individuals in highly affected municipalities. Diff. + in 2018 US Dollars. Diff. = Raw difference. p-value of *t*-test. Ho: mean difference = 0.

We performed similar descriptive analyses as above but split the data by mental health status. This is displayed in Table (A.1), which shows individual characteristics (sex, ethnicity, age, marital status, education, employment, and socioeconomic status) between those with higher and lower risk of developing mental health disorders across both periods. Overall, a higher proportion of women, individuals between 36 and 65 years of age, from a mestizo ethnicity, married, with a lower education background, and employed were found among those with a tendency to develop mental health problems. An interesting descriptive finding is that the proportion of people with harmful alcohol consumption was higher among those with no mental health disorders.

### 4.2 Results for the causal effect of conflict violence on mental health

Table (2) presents the estimation results for the effect of conflict-related violence on mental health. The second and third columns show the results from our DiD approach, with and without the effect of alcohol use, following Equations (1), (2). The fourth and fifth columns show the results from combining DiD and IV, according to Equations (3) and (4). All these specifications control for time-variant observed confounders. Across all these model specifications, we consistently found null effects.

Regarding the results from the IV first stage, Table (A.2) shows two models, one that controls only for socioeconomic and household conditions, and a second that also incorporates the effect of harmful alcohol use. In both models, the coefficients associated with the IV indicate that, for each increase of 1 ha of coca produced, the likelihood of being highly exposed to conflict-related violent events decreased by 0.002. This implies an inverse correlation between coca production and conflict-related violence, whereby increased coca production is associated with a lower chance of experiencing conflict-related violence. The inclusion of the harmful alcohol consumption indicator does not change the magnitude of this effect. These estimates are statistically significant at the 99% level. The relevance of coca production in predicting individual conflict exposure is further validated by statistics related to the under- and weak-identification tests, which are greater than the critical value of 16.38 (Stock & Yogo, 2005, pp. 80–108) and the commonly accepted threshold of 10. Moreover, we did not find any statistically significant evidence of coca production being associated with mental health disorders, as shown in Table (A.3). This supports our argumentation that the coca instrument is relevant and strong, and that coca production affects mental health only through individual exposure to conflict violence.

The results from the reverse causality test do not support the presence of an effect running from municipal conflict violence intensity to subsequent levels of coca production during our study period (see Table (A.5) in the ESM). The individual point estimates of the coca production lead terms are very small in size and not statistically significant at conventional levels, whilst an F-test cannot reject the joint null hypothesis of the estimated lead coefficients for coca production in column 2 being equal to zero. These results provide further reassurance about the validity of our coca production instrumental variable.

### 4.3 Results: does alcohol use mediate the effect of conflict violence on mental health?

We report the results of the causal mediation analysis exploring the "self-medication" hypothesis in Table (A.4). The estimates indicate that harmful alcohol consumption did not play a significant role in the relationship between conflict-related violence and mental health. This can be explained by our finding that conflict violence did not impact harmful alcohol use. Thus, we find no evidence supporting the "self-medication" hypothesis. It is worth noting that results from this specific mediation analysis should not be interpreted in causal terms, given the lack of a time-varying IV for the mediators, and harmful alcohol use.

### 4.4 Results from additional analysis

In a related, exploratory analysis, we investigated the "daily stressors" hypothesis, the results of which are presented in Figure (A.4) and Tables (A.6-A.10). We found that sequentially adding proxy variables for demographic, socioeconomic circumstances and household conditions left the estimated coefficient associated with post-accord conflict violence largely unchanged. We also found that being married decreases the probability of developing harmful alcohol consumption and mental health disorders by around 0.11.

## 5 Discussion

Previous research on Colombia has suggested that people exposed to violence related to the country's armed conflict have worse mental health than those not (or less) exposed to it. In this study, we added to this evidence base, by reporting more nuanced results. First, while there was an overall trend of worsening mental health among Meta residents in the four years between 2014 and 2018, we found no evidence that people living in municipalities with high levels of conflict violence had a different likelihood of developing mental health disorders in subsequent years, compared to people living in municipalities less affected or unaffected by conflict violence. Second, we found no evidence of a possible alcohol consumption pathway mediating any relationship between conflict violence exposure and mental health. Third, we found suggestive evidence that being married was protective against developing mental health disorders.

Our study is, to the best of our knowledge, the first to investigate in a causal framework the extent to which changes in violence exposure – using the 2016 Colombian peace agreement as a natural experiment – affect individual mental health, and to use formal mediation analysis to shed light on the role of alcohol consumption as a potential mediator between conflict violence exposure and mental health. Our analyses account for key potential sources of endogeneity, using an analytical strategy that combines the strengths of DiD and instrumental variables analyses. With any instrumental variables analysis, it is crucial to scrutinise the underlying assumptions of validity and relevance. Contextual and statistical evidence support our choice of IV. Results from the first-stage estimations confirm the relevance and strength of the IV, while further tests based on conditional correlations support its exogeneity. This analysis confirms our hypothesis that the FARC had firm, historically established territorial control over the areas selected to serve as their main coca production bases, which remained well-protected and mostly uncontested, unlike other regions of the Meta departamento.

There may exist some potential unobservable pathways directly affecting people's mental health that are correlated with coca production. One of the main candidates could be coca consumption. However, in Colombia, cocaine has mainly been produced to be exported: while Colombia is the main producer of this illegal drug, it sits at the 33rd position worldwide for cocaine consumption (UNDOC, 2019, 2023). This is also confirmed by the United Nations estimates of the prevalence of cocaine use, showing that its consumption among people aged 12–65 is relatively low in relation to coca production. Thus, coca consumption as an unobserved pathway is not likely to threaten the validity of our instrumental variable.

When comparing the results to the previous literature, we find some important, at times surprising differences. We found a general reduction in mental health between 2014 and 2018, a period that included the 2016 peace accord. Through a "trauma pathway", we would expect a person's mental health to improve post-peace accord due to the reduced trauma generated by lower levels of violence, including through reduced violence suffered by others, such as family members or peers. There is credible evidence of reduced mental stress among pregnant women and mothers stemming from decreases in violence in the communities where they lived (Masi, Hawkley, Harry Piotrowski, & Pickett, 2007; Ncube, Enquobahrie, Albert, Herrick, & Burke, 2016). Yet a trauma pathway also includes the possibility of mental health being negatively affected by the lagged effect of violence on trauma. For example, in the case of the Bosnia and Herzegovina conflict, one study found that individuals who experienced war trauma had worse mental health six years after the end of the conflict (Bratti et al., 2015). While this may conceivably apply to the Colombian context, a thorough exploration would require a longer time series of data than we have available to test for the presence of such dynamic (lagged) effects.

Our mediation analysis results are also in contrast to a previous study for Colombia, which pointed to increased alcohol consumption among conflict-affected individuals as a mechanism for mediating the effect of conflict on mental health (Londoño et al., 2012). However, the latter study only examined unconditional associations under a cross-sectional design for the year 2011, which will have severely limited its ability to control for various sources of biases (as discussed above) that may be confounding the hypothesised relationships. By contrast, in our study, we investigate the potential alcohol consumption pathway through formal mediation analysis, in which we control for observed and time-invariant unobserved heterogeneity across individuals exposed to different intensities of conflict violence. Our results are more in line with the results from a recent systematic literature review, which found only weak evidence of individuals exposed to conflict being more prone to hazardous alcohol consumption (Lo et al., 2017).

In this paper, we have also explored other possible pathways that can be behind our null findings on the effect of conflict violence on mental health. The "daily-stressors pathway" would suggest that mental health is less affected by exposure to conflict violence itself, and more so by the social and economic conditions that people find themselves in daily (Miller & Rasmussen, 2010). However, finding that certain individual characteristics do exhibit an independent association with mental health outcomes (e.g. people who were married were less likely to develop mental health disorders, as also found in other contexts (Roberts & Browne, 2011)), provides some evidence that marital status might be an important driver of mental health in the context of conflict and conflict de-escalation. This pathway is further supported by the specific features of the post-peace accord context in Colombia. Before their demobilisation, the FARC essentially created a parallel state, with their institutions and the provision of services. For many years and across different regions of the country (including in the Meta departamento), the FARC provided local communities with services (e.g. basic healthcare, security), opportunities to earn a living (informal jobs), and an officially unrecognised, yet established institutional framework (Arjona, 2016; Justino, 2019). The demobilisation of the FARC in areas where it used to be the dominant actor led to the dismantling of these institutional frameworks, resulting in uncertain prospects and a vacuum that was not immediately filled by the Colombian government, including regarding the provision of security and other basic public services (Piccone, 2019). This, in turn, may have hampered any mental health benefits arising from reduced individual exposure to violence, which highlights the need for effective peace agreements to include, beyond the cessation of hostilities, the timely implementation of social and economic policies targeted at those populations highly exposed to pre-existing conflict violence, alongside health interventions that address the trauma generated by direct exposure to violence (Miller & Rasmussen, 2010).

Some limitations of our study must be borne in mind when interpreting the findings. First, our baseline individual-level data were collected retrospectively and, therefore, some measurement errors may be present. Nonetheless, analyses detailed elsewhere (Moreno-Serra, Anaya-Montes, León-Giraldo, & Bernal, 2022) have shown that individual recall error in our data is not correlated with living in high or low-conflict intensity areas, which could have biased our estimates. Another potential explanation for the null effects found in our study is that the analysis might be underpowered. The internal validity of our analysis is supported by: i) a sample size of 1309 observations for each period, ii) a relatively small number of independent variables included in the regression models (around 14–16 variables), and iii) coca production (our IV) being a strong predictor of conflict-related violence. Yet we cannot completely rule out the possibility that a non-zero effect exists. Given the relatively wide confidence interval estimated for our coefficient of interest, which ranges between −0.5 and 0.4 (DiD-IV model in Table 2), it is possible that with our data we are unable to detect effects that may lie within such range. Although a post-hoc power analysis could give valuable insights into this issue, the methodological work to simulate data that reflect our specific setting outweighs the potential benefits of doing it, and it is outside the scope of this paper. Our analysis does support, however, the absence of relatively large mental health effects that are driven directly by different degrees of exposure to conflict violence, which have been suggested (at least implicitly) in previous analyses for Colombia. For example, descriptive studies have concluded that 10.8% of the people living in Colombian municipalities constantly affected by conflict experienced mental health disorders (Gómez-Restrepo et al., 2016), and that the prevalence of somatisation disorder and depression was 9 percentage points higher in a municipality with a high level of conflict-related violence compared to another municipality with low conflict-related violence (Londoño et al., 2012).

**Table 2:** Regression results. DiD and IV estimations.

	DID	DID, Alcohol use	DID-IV	DID-IV, Alcohol use
Conflict violence in 2018	−0.015	−0.015	−0.041	−0.044
	[-0.078,0.048]	[-0.079,0.049]	[-0.484,0.403]	[-0.428,0.339]
***Mediator***				
Positive AUDIT		−0.064		−0.065
		[-0.141,0.013]		[-0.146,0.016]
***Individual Characteristics***				
Age: 36–65 years	0.004	0.001	0.004	0.001
	[-0.097,0.105]	[-0.098,0.100]	[-0.102,0.109]	[-0.102,0.103]
Age: >66 years	0.055	0.053	0.056	0.053
	[-0.123,0.234]	[-0.124,0.230]	[-0.124,0.235]	[-0.123,0.230]
Above Secondary	−0.055	−0.058	−0.050	−0.052
	[-0.249,0.139]	[-0.254,0.137]	[-0.249,0.150]	[-0.251,0.147]
Single	−0.025	−0.026	−0.023	−0.024
	[-0.134,0.085]	[-0.136,0.083]	[-0.142,0.096]	[-0.141,0.092]
Married	−0.110**	−0.114***	−0.111**	−0.115***
	[-0.195,-0.026]	[-0.200,-0.028]	[-0.196,-0.025]	[-0.202,-0.028]
Paid job	−0.170	−0.164	−0.166	−0.159
	[-0.529,0.189]	[-0.513,0.185]	[-0.531,0.199]	[-0.510,0.192]
No-work paid	0.056	0.060	0.052	0.055
	[-0.335,0.448]	[-0.320,0.440]	[-0.345,0.449]	[-0.333,0.443]
Unpaid job	−0.152	−0.151	−0.149	−0.147
	[-0.537,0.232]	[-0.527,0.226]	[-0.533,0.235]	[-0.521,0.228]
***Household conditions***				
HAI:q2	0.014	0.016	0.014	0.017
	[-0.081,0.108]	[-0.080,0.112]	[-0.082,0.110]	[-0.080,0.114]
HAI:q3	0.008	0.010	0.009	0.011
	[-0.089,0.105]	[-0.088,0.109]	[-0.092,0.110]	[-0.090,0.113]
HAI:q4	0.077	0.081	0.077	0.081
	[-0.036,0.190]	[-0.035,0.196]	[-0.038,0.192]	[-0.037,0.198]
Household expenditure^a^	0.103*	0.105*	0.100	0.102
	[-0.013,0.218]	[-0.012,0.221]	[-0.036,0.235]	[-0.034,0.237]
2018	0.179***	0.177***	0.190**	0.190**
	[0.127,0.230]	[0.125,0.228]	[0.006,0.374]	[0.017,0.362]
Observations	2618	2618	2618	2618
Kleibergen-Paap (KP) LM statistic+			489	489
p-value of KP LM statistic			2.61e-108	2.19e-108
Kleibergen-Paap Wald F++			772	772

Notes: Bootstrapped 95 confidence intervals in brackets (1000 reps). Reference categories: young adults, other marital status, does not know job status and, first quartile of household conditions. ^a^Monthly expenditure, expressed in 2018 thousand USD dollars. +Under identification test. ++Weak identification test. *p < 0.1, **p < 0.05, ***p < 0.01.

Second, our dataset covers a relatively short period, hence preventing us from examining possibly lagged impacts of conflict violence (and its reduction) on mental health. The omission of these lagged effects is likely to cause an underestimation of the effect of conflict-related violence on mental health. Third, our proxy indicators of potential mental health disorders and harmful alcohol consumption were constructed from screening tools and do not constitute clinical diagnostic instruments (Beusenberg et al., 1994). Nevertheless, our mental health indicator captures a person's tendency to develop a wide spectrum of mental health disorders, encompassing psychiatric, somatisation and eating disorders.

A final limitation is that we were unable to adopt an instrumental variable approach for our alcohol consumption mediation analysis. While we can leverage individual changes in potentially harmful alcohol consumption between 2014 and 2018, allowing us to control for variations among individuals in observed and unobserved time-invariant characteristics, we can only account for individual differences in time-varying factors through the inclusion of a set of socioeconomic and demographic observable covariates. Consequently, some unobserved time-varying confounding may still influence our mediation analysis results. Variables that have been used in other contexts as instruments for individual alcohol consumption were not available as suitable time-varying information in our case, e.g. availability of alcohol sales outlets (Mentzakis, Suhrcke, Roberts, Murphy, & McKee, 2013) or intensity of alcohol advertisement in the area of residence (Mentzakis, Roberts, Suhrcke, & McKee, 2016). Hence, with our data, we cannot rule out the possibility that our result regarding the absence of a mediating role of alcohol consumption might be driven by remaining confounding. This implies that results from the mediation analysis cannot be interpreted in causal terms, but rather as an exploratory exercise. Future research to investigate this issue further seems warranted. Another direction for future research is related to investigating which mental health disorder (anxiety, depression, post-traumatic stress disorder, disruptive behaviour, dissocial disorders, etc.) is the most affected by armed conflict, and whether this affects other expressions of violence, such as family violence.

Our results give rise to two key recommendations for mental health policy in Colombia. The first stems from the observation that the current public approach to preventing alcohol and drug consumption does not treat victims of conflict as high-risk populations for developing harmful alcohol consumption or mental health disorders (Minsalud, 2018). Our paper demonstrated that mental health has deteriorated over time among the Colombian population. Despite this not being a direct consequence of conflict-related violence, it calls for more attention to promote mental healthcare, both at the national and sub-national levels. Second, our analysis exploring the "daily stressors hypothesis" also highlights that improvement of socioeconomic conditions could also be considered when designing policies to reduce the health burden of victims of conflict. Our evidence supports the view that the planning and introduction of social and economic policies in areas affected by conflict violence, to rebuild state institutions and reinstate the provision of effective public services including healthcare, should be integral to – and happen in tandem with – the implementation of ceasefires and peace agreements in conflict settings. This seems crucial to protect the health and welfare of conflict-affected populations from the consequences of past violence, as well as from any unintended consequences arising from the institutional and socioeconomic changes taking place in the post-conflict environment.

## Funding statement

This research was funded by the UK Medical Research Council (MRC), Economic and Social Research Council (ESRC), DFID, and The Wellcome Trust, through the Joint Health Systems Research Initiative (Grant Number MR/R013667/1). The funders of the study had no role in the study design, data collection, data analysis and interpretation, or writing.

For the purpose of open access a Creative Commons Attribution (CC BY) licence is applied to any Author Accepted Manuscript version arising from this submission.

## Conflict of interest disclosure

The authors declare that they have no conflict of interest.

## Ethics approval statement

The War and Peace project, including the CONPAS survey and all aspects of its application, received ethical (IRB) approval from both the Universidad de los Andes (Colombia) and the University of York (UK).

## CRediT authorship contribution statement

**Andrea Salas-Ortiz:** Writing – review & editing, Writing – original draft, Software, Methodology, Formal analysis. **Rodrigo Moreno-Serra:** Writing – review & editing, Supervision, Methodology, Conceptualization. **Noemi Kreif:** Writing – review & editing, Software, Methodology. **Marc Suhrcke:** Writing – review & editing. **German Casas:** Writing – review & editing.

## Data Availability

The dataset analysed in this study is available on reasonable request.

## Footnotes

1 Throughout the paper, we will use the terms "peace agreement", "peace accord" and "ceasefire accord" inter-changeably.

2 A broader description of this process is described in (Buitrago & Moreno-Serra, 2021).

3 Meta is one of the 32 departamentos of Colombia, i.e. provinces.

4 Number of annual violent events in each municipality divided by the annual number of inhabitants in that municipality and multiplied by 100,000.

5 This trend was due to explicit and implicit, unilateral, and bilateral, ceasefire declarations by the government and/or the FARC to support the Havana peace talks that started in 2012. These talks set the foundation for the definitive 2016 peace agreement.

6 Note that *d*_*m,* 2014_ is time-constant variable, resulting in repeating the same observation for each municipality m for both time periods.

7 Such as, gypsies, raizal del archipiélago, palenquero de San Basilio, black, mulato, or afro-Colombian.

8 For 2014, the first component reported an eigenvalue of 10.24 and explained 16.26% of the total variance. For 2018, the eigenvalue of the first component was 6.52 and explained 25.28% of the total variance.

9 For this, we used the GDP inflation deflator from the World Bank and a US-Colombian peso exchange rate of 1 US-dollar = 2955.704 Colombian pesos.

10 The null hypothesis being that the difference between groups is zero.
