# Supplementary material for: The effect of conflict-related violence intensity and alcohol use on mental health: The case of Colombia
Source: SSM Popul Health. 2024 Feb 8;25:101626. doi: 10.1016/j.ssmph.2024.101626 (PMC10884503; doi:10.1016/j.ssmph.2024.101626)
Supplement: Multimedia component 2 [file mmc2.docx]

The effect of conflict-related violence intensity and alcohol use on mental health: the case of Colombia

space

Electronic Supplementary Material

#

# A.1 Instrumental variable estimation

We depict this setting in Figure 3, where we denote our instrumental variable for conflict-related violence as *z_mt_*, defined as the annual production of coca (in hectares), in municipality *m* in year *t*. Consistent IV estimates must meet two validity assumptions. First, the instrumental variable, *z,* should be a relevant and strong predictor of *d*_2014_∗*t*_2018_ *(Cov:z,d* ̸= *0)* (depicted by the thick black arrow from *z* to *d*). Second, it should not be correlated with *ϵ*, i.e. *(Cov:z, ϵ* = 0*)*. This is known as the *exclusion restriction* assumption and implies two conditions. First, that *z_mt_* is exogenous to *y_imt_* so that, conditional on the controls *X_imt_*, *z_mt_* ⊥ *y_imt_*. In other words, there are no unobserved confounders that affect both the outcome and the instrument. Second, that *z_mt_* only affects *y_imt_* through *d_imt_*, i.e. *z_mt_* must not have a direct influence on *y_imt_* (represented by orange dotted lines, from *u* to *z* and from *z* to *y*).

Figure 3: Instrumental variable estimation

*IV*

(

*z*

)

*Conflict*

(

*d*

)

*Alcohol*

(

*m*

)

*MentalHealth*

(

*y*

)

*(Un)observables (u)*

The two-step least squares (2SLS) model involves regressing the endogenous variable on the IV controlling for other potential confounding factors, as well as individual and time fixed effects. In this first stage, we regress *d_imt_*, defined as *d*_2014_ ∗ *t*_2018_, against *z_mt_* to capture its exogenous variation, while also controlling for the exogenous variables *X_imt_* and time and municipality fixed effects, as below:

*dm,*2014 ∗ *t*2018 = *α* + *ϕzmt* + *γXimt* + *θt* + *θi* + *ωimt* (3)

The fitted value from this equation, $\hat{d_{imt}}$, captures the exogenous variation in conflict-related violence, e.g, that is not correlated with *ϵ_imt_*.

In the second stage,$\hat{d_{m,2014*t2018}}$ replaces *d_m,_*_2014_ ∗ *t*_2018_, so that:

$y_{imt}=\alpha+\hat{\tau d_{m,2014}*t_{2018}}+\gamma X_{imt}+\theta_{t}+\theta_{i}+\epsilon_{imt}$ (4)

# A.2 Mediation analysis

Mediation analysis aims to assess the causal mechanisms along the pathway between a treatment (in our case: conflict exposure) and a given outcome (in our case: mental health), disentangling the total effect of treatment into an indirect effect that operates via one or several observable intermediate factors (mediators) and a direct effect that reflects any impact not captured by the observed mediators. Here, we focus on one mediator only, heavy alcohol consumption.

To introduce the mediation approach, we define *y_it_*(*d*) to be the hypothetical value of the outcome (from here on: potential outcome) that would have occurred if *D_i_* had been set to *d*, and let *y_it_*(*d,m*) be the potential outcome that would have occurred if *D_i_* had been set to *d* and *M_it_* had been set to *m*. Similarly, let *m_it_*(*d*) be the potential value of the mediator if *D_i_* had been set to *d*.

The natural *direct* effect (NDE)^^[[1]](#footnote-1)^^ is defined as:

*NDE* = E[*y_it_*(1*,m_it_*(0)) − *y_it_*(0*,m_it_*(0))]

and the natural *indirect* effect (NIE) is defined as

*NIE* = E[*y_it_*(1*,m_it_*(1)) − *y_it_*(1*,m_it_*(0))]

and, the total direct effect (TE) is

*TE* = *NDE* + *NIE*

We estimate these quantities within our DiD-IV design and using the following three equations. For notational simplicity, below we present the equations for the DiD analysis, which are amended with instrumental variables the way described in sub-section 2.4.

The first equation captures the TE, and is estimated as before:

$y_{imt}=\alpha^{1}+\tau_{D}^{1}d_{m,2014}*t_{2018}+\gamma_{X}^{1}X_{imt}+\theta_{t}^{1}+\beta_{i}^{1}+\epsilon_{imt}^{1}$ (6)

For the estimation of the NIE, a second equation is necessary, where (6) is expanded by also adding the mediator on the right-hand side.

$y_{imt}=\alpha^{2}+\tau_{D}^{2}d_{m,2014}*t_{2018}+\delta_{M}^{2}m_{imt}+\gamma_{X}^{2}X_{imt}+\theta_{t}^{2}+\beta_{i}^{2}+\epsilon_{imt}^{2}$ (7)

Finally, for the estimation of NDE, a third equation is necessary, where the mediator, the mediator (*m*), harmful alcohol use, is the dependent variable, modelled as a function of conflict exposure and control variables:

$m_{imt}=\alpha^{3}+\tau_{D}^{3}d_{m,2014}*t_{2018}+\gamma_{X}^{3}X_{imt}+\theta_{t}^{3}+\beta_{i}^{3}+\epsilon_{imt}^{3}$ (8)

The coefficients have over-scripts to denote the specific equation they refer to (Equation 1, 2 or 3), and the under-scripts denote the corresponding explanatory variable. The TE is captured by *τ_D_*^1^, the NDE by *τ_D_*^2^ and the NIE is the product of *δ_M_*^2^ and *τ_D_*^3^.

For the robust estimation of these effects, three assumptions are required: 1) no unmeasured confounding between the exposure to conflict violence and mental health (outcome); 2) no unmeasured confounding between harmful alcohol use (the mediator) and the outcome, and

3) no unmeasured confounding between the exposure to conflict violence and the mediator. Assumptions 1) and 3) are likely to hold given our DiD-IV estimation strategy. However, this is not the case for assumption 2) since there might be confounders affecting simultaneously the mediator and the outcome. This could be tackled via an IV for harmful alcohol use. However, no time-varying data on a relevant, strong and likely exogenous IV was available for this purpose.

Thus, the NIE cannot be interpreted in causal terms.

Figure A.1: Conflict-related violence rates among lightly or unexposed and highly exposed* municipalities before and after the 2016-peace accord


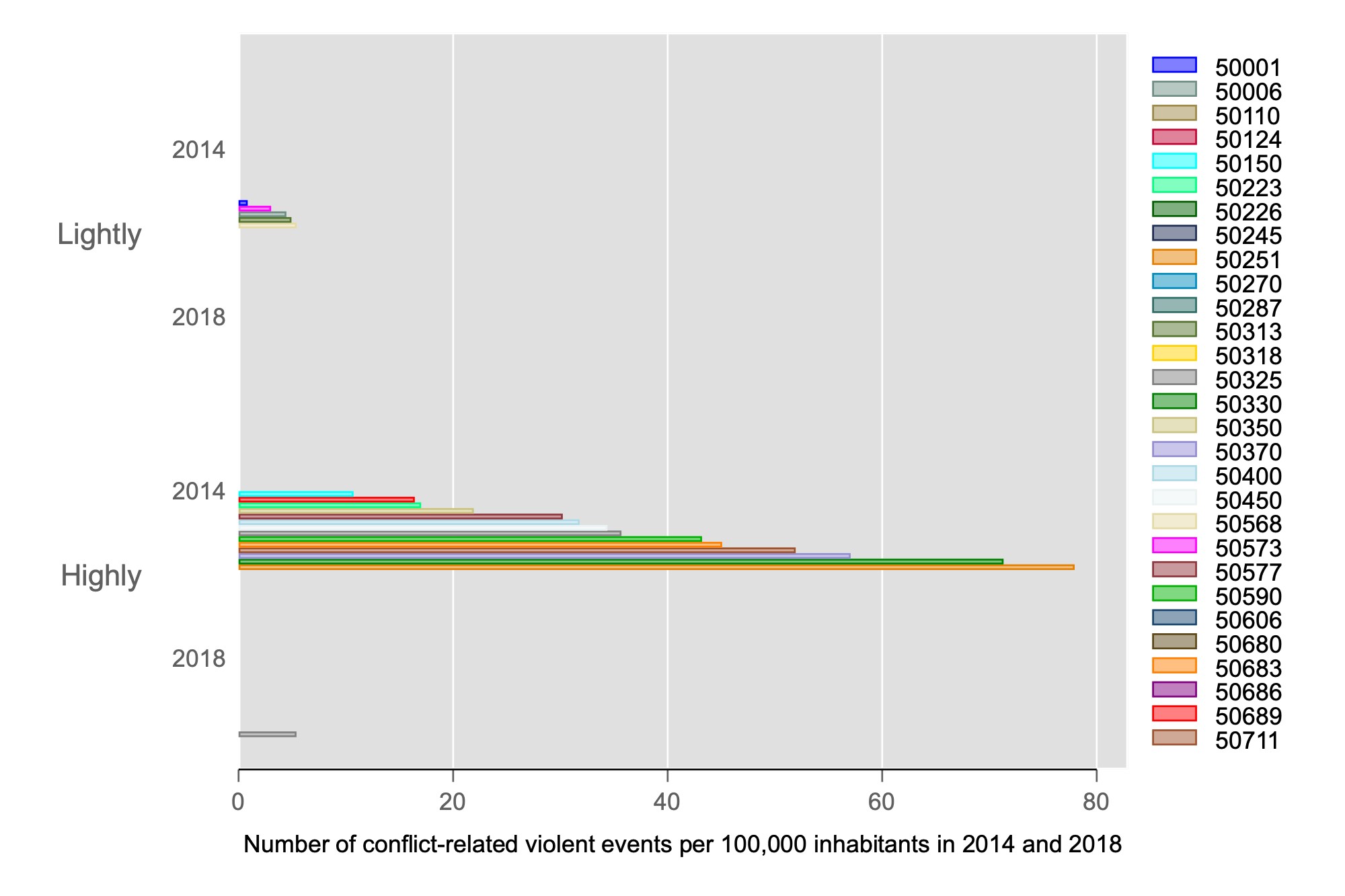


Note :*Classified according to the median 2014-conflict rate. Legend identifies Meta’s municipalities

Figure A.2: Distribution of the number of total violent events by 100,000 inhabitants in 2014


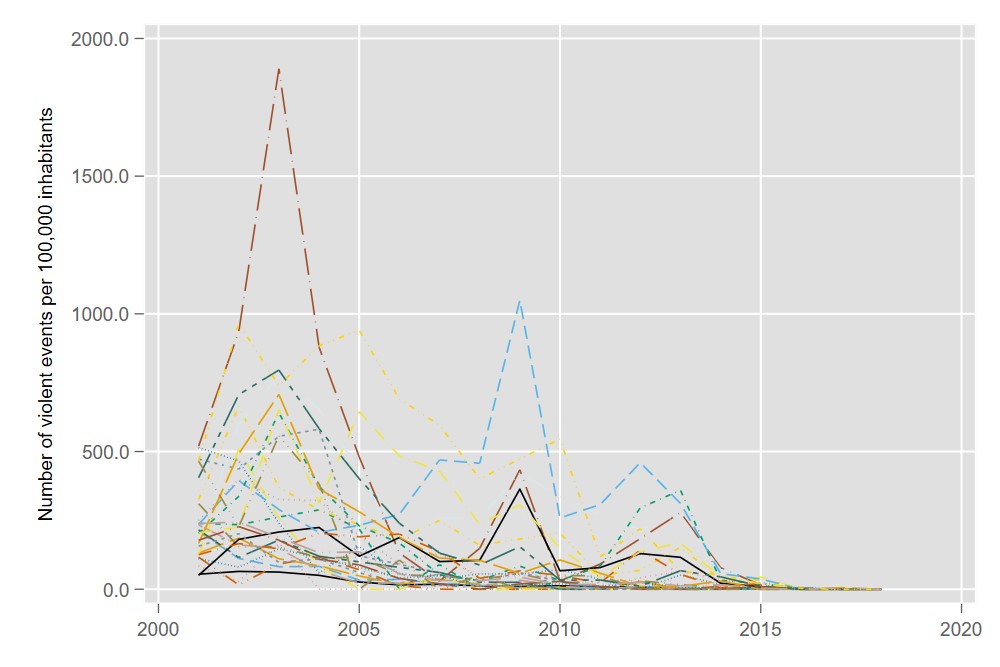


Note: Each line represents a municipality of Meta departamento

Figure A.3: Distribution of the number of total violent events by 100,000 inhabitants in 2014


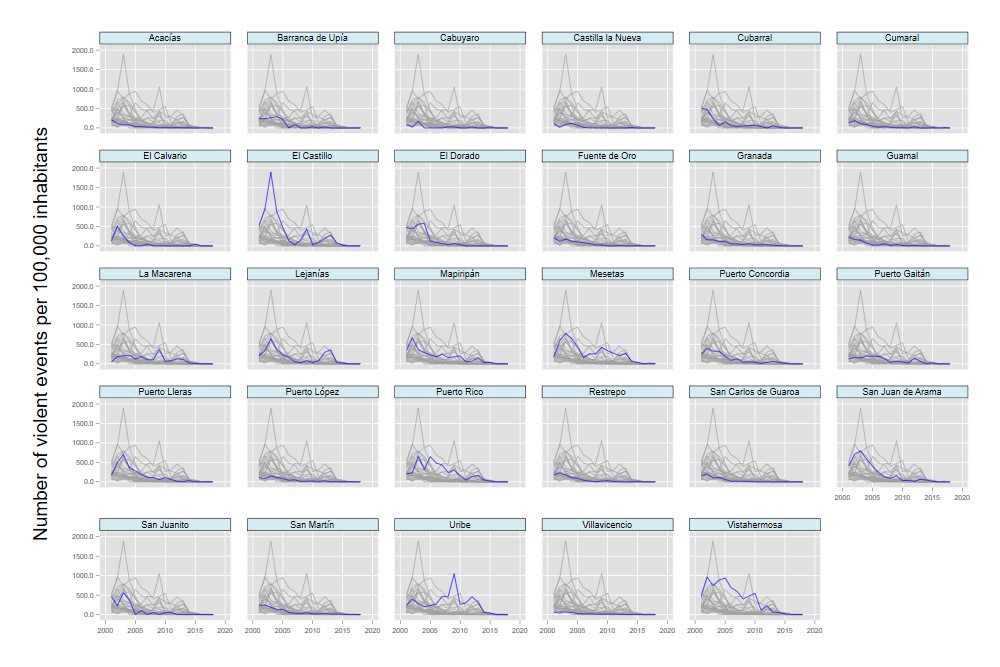


Table A.1: Average characteristics of individuals given mental health status over periods.

|  | **Pre-accord (2014)** | | | **Post-accord (2018)** | | |
| --- | --- | --- | --- | --- | --- | --- |
|  | **SRQ=0** | **SRQ=1** | **p-val** | **SRQ=0** | **SRQ=1** | **p-val** |
| Covariates |  |  |  |  |  |  |
| Men | 0.49 | 0.31 | 0.00 | 0.55 | 0.31 | 0.00 |
| ***Age*** |  |  |  |  |  |  |
| Below 35 years old | 0.39 | 0.30 | 0.01 | 0.34 | 0.24 | 0.00 |
| 36-65 years old | 0.52 | 0.59 | 0.06 | 0.53 | 0.59 | 0.03 |
| Above 66 years old | 0.09 | 0.11 | 0.32 | 0.13 | 0.17 | 0.06 |
| ***Ethnicity*** |  |  |  |  |  |  |
| White | 0.36 | 0.32 | 0.17 | 0.35 | 0.36 | 0.66 |
| Indigenous | 0.04 | 0.06 | 0.25 | 0.04 | 0.06 | 0.33 |
| Mestizo | 0.43 | 0.43 | 1.00 | 0.45 | 0.39 | 0.03 |
| Other | 0.16 | 0.19 | 0.26 | 0.15 | 0.19 | 0.07 |
| ***Marital Status*** |  |  |  |  |  |  |
| Single | 0.14 | 0.07 | 0.00 | 0.09 | 0.06 | 0.04 |
| Married | 0.66 | 0.61 | 0.16 | 0.66 | 0.56 | 0.00 |
| Other Status | 0.20 | 0.33 | 0.00 | 0.25 | 0.38 | 0.00 |
| Education |  |  |  |  |  |  |
| None or below sec. | 0.83 | 0.87 | 0.18 | 0.78 | 0.85 | 0.00 |
| > secondary school | 0.17 | 0.13 | 0.18 | 0.22 | 0.15 | 0.00 |
| ***Job Status*** |  |  |  |  |  |  |
| Paid job | 0.68 | 0.63 | 0.14 | 0.64 | 0.53 | 0.00 |
| No-work paid | 0.03 | 0.03 | 0.92 | 0.03 | 0.03 | 0.98 |
| Unpaid job | 0.29 | 0.33 | 0.17 | 0.32 | 0.42 | 0.00 |
| Unknown job | 0.01 | 0.01 | 0.34 | 0.01 | 0.01 | 0.31 |
| ***Household characteristics*** | |  |  |  |  |  |
| Average expenditure^a^ | 277.69 | 293.74 | 0.24 | 308.57 | 309.82 | 0.92 |
| Asset Index: q1 | 0.25 | 0.28 | 0.33 | 0.26 | 0.24 | 0.55 |
| Asset Index: q2 | 0.24 | 0.29 | 0.14 | 0.23 | 0.29 | 0.01 |
| Asset Index: q3 | 0.24 | 0.29 | 0.14 | 0.25 | 0.25 | 0.91 |
| Asset Index: q4 | 0.27 | 0.15 | 0.00 | 0.27 | 0.22 | 0.05 |
| AUDIT+ | 0.16 | 0.12 | 0.07 | 0.13 | 0.10 | 0.07 |
| Observations | 1,309 |  |  | 1,309 |  |  |

Notes: SRQ=0: individuals not at risk of developing mental health disorders. SRQ=1: individuals at risk of developing mental health disorders. AUDIT+=People with harmful alcohol consumption. $^a$ Monthly expenditure, expressed in 2018 thousand USD dollars. p-val of t-test. Ho: mean difference=0


Table A.2: DID-IV. Mediation Analysis. First stage results.

|  | D\|X^a^ | D\|M,X |
| --- | --- | --- |
| *Instrumental variable* |  |  |
| Coca Production Hectares | -0.002*** | -0.002*** |
|  | [-0.002,-0.002] | [-0.002,-0.002] |
| ***Mediator*** |  |  |
| Positive AUDIT |  | 0.010 |
|  |  | [-0.034,0.054] |
| ***Individual Characteristics*** |  |  |
| Age: 36-65 years | -0.028 | -0.027 |
|  | [-0.077,0.022] | [-0.077,0.022] |
| Age: >66 years | -0.030 | -0.030 |
|  | [-0.113,0.054] | [-0.113,0.054] |
| Above Secondary | 0.040 | 0.041 |
|  | [-0.027,0.107] | [-0.026,0.107] |
| Single | 0.044 | 0.044 |
|  | [-0.024,0.113] | [-0.025,0.113] |
| Married | 0.003 | 0.003 |
|  | [-0.038,0.043] | [-0.038,0.044] |
| Paid job | 0.052 | 0.051 |
|  | [-0.115,0.219] | [-0.116,0.218] |
| No-work paid | 0.036 | 0.036 |
|  | [-0.161,0.232] | [-0.161,0.232] |
| Unpaid job | 0.044 | 0.044 |
|  | [-0.125,0.213] | [-0.125,0.213] |
| ***Household conditions*** |  |  |
| HAI: q2 | -0.011 | -0.012 |
|  | [-0.058,0.035] | [-0.058,0.035] |
| HAI: q3 | -0.019 | -0.020 |
|  | [-0.071,0.032] | [-0.071,0.032] |
| HAI: q4 | -0.005 | -0.006 |
|  | [-0.061,0.051] | [-0.061,0.050] |
| Household expenditure^a^ | -0.035 | -0.035 |
|  | [-0.102,0.032] | [-0.102,0.032] |
| 2018 | 0.111*** | 0.111*** |
|  | [0.093,0.128] | [0.093,0.129] |

Notes: Bootstrapped 95 confidence intervals in brackets (1,000 reps). Reference categories: young adults, other marital status, does not know job status and, first quartile of household conditions. ^a^Monthly expenditure, expressed in 2018 thousand USD dollars. +Under identification test. ++Weak identification test. * p<0.1, ** p<0.05, *** p<0.01


Table A.3: Association between mental health and cocaine production at baseline

|  | **Individual-level controls: NO** | **Individual-level controls: YES** |
| --- | --- | --- |
| High cocaine production in 2014 (p50) | 0.022 | -0.002 |
|  | (0.023) | (0.028) |
| ***Individual characteristics*** |  |  |
| Age: 36-65 years |  | 0.019 |
|  |  | (0.024) |
| Age: >66 years |  | 0.006 |
|  |  | (0.045) |
| Above Secondary |  | 0.002 |
|  |  | (0.030) |
| Single |  | -0.161*** |
|  |  | (0.037) |
| Married |  | -0.096*** |
|  |  | (0.030) |
| Paid job |  | -0.111 |
|  |  | (0.143) |
| No-work paid |  | -0.105 |
|  |  | (0.160) |
| Unpaid job |  | -0.057 |
|  |  | (0.144) |
| ***Household conditions*** |  |  |
| HAI: q2 |  | 0.003 |
|  |  | (0.034) |
| HAI: q3 |  | -0.001 |
|  |  | (0.036) |
| HAI: q4 |  | -0.095*** |
|  |  | (0.036) |
| Household expenditure^a^ |  | 0.146** |
|  |  | (0.062) |
| Observations | 1,309 | 1,309 |

Notes: The dependent variable is high risk of developing mental health disorders. Reference categories: young adults, other marital status, does not know job status and, first quartile of household conditions. ^a^ Monthly expenditure, expressed in 2018 thousand USD dollars. * p<0.1, ** p<0.05, *** p<0.01. Standard errors in parentheses


Table A.4: DID-IV. Mediation Analysis. Second stage results.

|  | Y\|D,X | Y\|D,M,X | M\|D,X |
| --- | --- | --- | --- |
| Conflict violence in 2018 | -0.041 | -0.044 | -0.056 |
|  | [-0.484,0.403] | [-0.428,0.339] | [-0.565,0.453] |
| ***Mediator*** |  |  |  |
| Positive AUDIT |  | -0.065 |  |
|  |  | [-0.146,0.016] |  |
| ***Individual Characteristics*** | |  |  |
| Age: 36-65 years | 0.004 | 0.001 | -0.046 |
|  | [-0.102,0.109] | [-0.102,0.103] | [-0.137,0.045] |
| Age: >66 years | 0.056 | 0.053 | -0.032 |
|  | [-0.124,0.235] | [-0.123,0.230] | [-0.148,0.083] |
| Above Secondary | -0.050 | -0.052 | -0.036 |
|  | [-0.249,0.150] | [-0.251,0.147] | [-0.190,0.118] |
| Single | -0.023 | -0.024 | -0.020 |
|  | [-0.142,0.096] | [-0.141,0.092] | [-0.123,0.084] |
| Married | -0.111** | -0.115*** | -0.065** |
|  | [-0.196,-0.025] | [-0.202,-0.028] | [-0.124,-0.007] |
| Paid job | -0.166 | -0.159 | 0.108 |
|  | [-0.531,0.199] | [-0.510,0.192] | [-0.133,0.348] |
| No-work paid | 0.052 | 0.055 | 0.051 |
|  | [-0.345,0.449] | [-0.333,0.443] | [-0.337,0.439] |
| Unpaid job | -0.149 | -0.147 | 0.030 |
|  | [-0.533,0.235] | [-0.521,0.228] | [-0.223,0.284] |
| ***Household conditions*** |  |  |  |
| HAI: q2 | 0.014 | 0.017 | 0.039 |
|  | [-0.082,0.110] | [-0.080,0.114] | [-0.015,0.093] |
| HAI: q3 | 0.009 | 0.011 | 0.035 |
|  | [-0.092,0.110] | [-0.090,0.113] | [-0.026,0.096] |
| HAI: q4 | 0.077 | 0.081 | 0.061 |
|  | [-0.038,0.192] | [-0.037,0.198] | [-0.021,0.144] |
| Household expenditure^a^ | 0.100 | 0.102 | 0.031 |
|  | [-0.036,0.235] | [-0.034,0.237] | [-0.092,0.155] |
| 2018 | 0.190** | 0.190** | -0.009 |
|  | [0.006,0.374] | [0.017,0.362] | [-0.207,0.190] |
| Observations | 2,618 | 2,618 | 2,618 |
| Kleibergen-Paap (KP) LM statistic+ | 489 | 489 | 489 |
| p-value of KP LM statistic | 2.61e-108 | 2.19e-108 | 2.61e-108 |
| Kleibergen-Paap Wald F++ | 772 | 772 | 772 |

Notes: The NIE is the product of -0.065 times -0.056. Bootstrapped 95 confidence intervals in brackets (1,000 reps). Reference categories: young adults, other marital status, does not know job status and, first quartile of household conditions. ^a^Monthly expenditure, expressed in 2018 thousand USD dollars. +Under identification test. ++Weak identification test. * p<0.1, ** p<0.05, *** p<0.01


Table A.5: Weak reverse causality test

|  | **Model 1** | **Model 2** |
| --- | --- | --- |
| ***Coca production and leads*** |  |  |
| Coca Production Hectares | 0.0000 | 0.0008 |
|  | (0.0008) | (0.0012) |
| Coca Production Hectares (t+1) | 0.0008 | 0.0011+ |
|  | (0.0005) | (0.0006) |
| Coca Production Hectares (t+2) |  | -0.0007 |
|  |  | (0.0008) |
| ***Individual Characteristics*** |  |  |
| Age: 36-65 years | 0.0472 | 0.0473 |
|  | (0.0353) | (0.0348) |
| Age: >66 years | 0.0102 | 0.0115 |
|  | (0.0499) | (0.0496) |
| Above secondary | -0.1013* | -0.1024* |
|  | (0.0463) | (0.0467) |
| Single | -0.0681+ | -0.0765+ |
|  | (0.0359) | (0.0377) |
| Married | 0.0082 | 0.0084 |
|  | (0.0141) | (0.0139) |
| Paid job | -0.1213 | -0.1204 |
|  | (0.0875) | (0.0877) |
| No-work paid | 0.0346 | 0.0475 |
|  | (0.0954) | (0.0923) |
| Unpaid job | -0.1090 | -0.1072 |
|  | (0.0759) | (0.0761) |
| ***Household conditions*** |  |  |
| HAI: q2 | -0.0262 | -0.0255 |
|  | (0.0347) | (0.0344) |
| HAI: q3 | -0.0340 | -0.0338 |
|  | (0.0510) | (0.0506) |
| HAI: q4 | -0.0236 | -0.0223 |
|  | (0.0461) | (0.0456) |
| Household expenditure^a^ | 0.0001 | 0.0001 |
|  | (0.0001) | (0.0001) |
| 2018 | -0.2573+ | -0.2601+ |
|  | (0.1270) | (0.1280) |
| F-stat of joint test |  | 1.7 |
| p-value of F-stat |  | .201 |
| Observations | 2,618 | 2,618 |

Notes: Clustered errors at municipality in parenthesis. t+1 means 2015 and 2019, t+2 means 2016 and 2020. No education, another marital status, does not know job status and the first quartile of household asset index (HAI) used as reference categories. ^a^Monthly expenditure, expressed in 2018 thousand USD dollars. + p<0.1, * p<0.05, ** p<0.01, *** p<0.001


Figure A.4: DiD-IV Second stage results for TE, NIE, and NDE equations

11


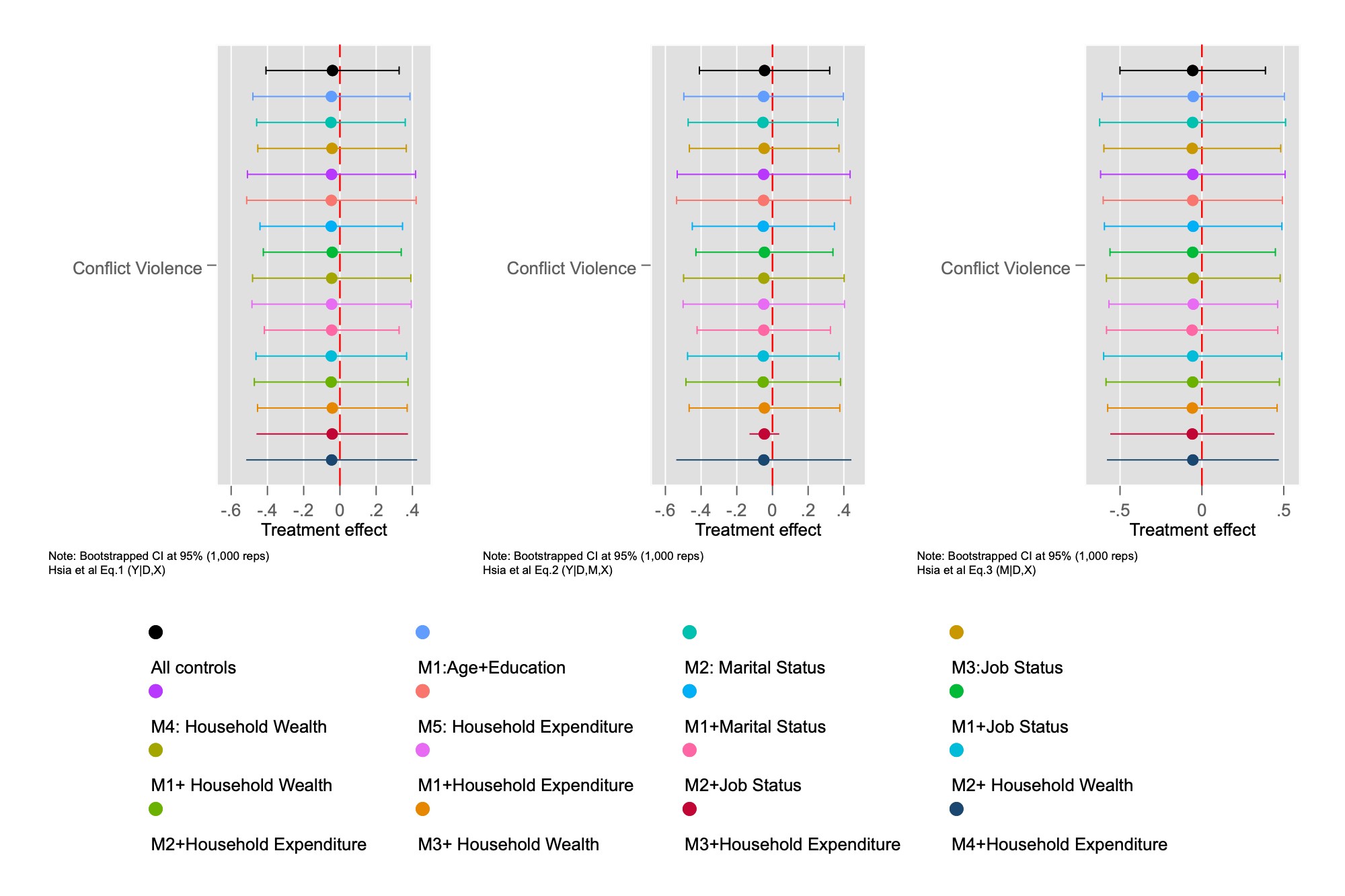


Note: Only the treatment coefficient,
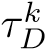
 with *k* =1*,*2*,*3, is displayed

Table A.6: DiD-IV first-stage results for TE equation


|  | M0 | M1 | M2 | M3 | M4 | M5 | M6 | M7 | M8 | M9 | M10 | M11 | M12 | M13 | M14 | M15 | M16 |
| --- | --- | --- | --- | --- | --- | --- | --- | --- | --- | --- | --- | --- | --- | --- | --- | --- | --- |
| ***IV*** |  |  |  |  |  |  |  |  |  |  |  |  |  |  |  |  |  |
| Coca Production | -0.001*** | -0.001*** | -0.001*** | -0.001*** | -0.001*** | -0.001*** | -0.001*** | -0.001*** | -0.001*** | -0.001*** | -0.001*** | -0.001*** | -0.001*** | -0.001*** | -0.001*** | -0.001*** | -0.001*** |
|  | (0.000) | (0.000) | (0.000) | (0.000) | (0.000) | (0.000) | (0.000) | (0.000) | (0.000) | (0.000) | (0.000) | (0.000) | (0.000) | (0.000) | (0.000) | (0.000) | (0.000) |
| ***Age and Educ.*** |  |  |  |  |  |  |  |  |  |  |  |  |  |  |  |  |  |
| Age: 36-65 years |  | -0.007 |  |  |  |  | -0.008 | -0.006 | -0.005 | -0.008 |  |  |  |  |  |  | -0.005 |
|  |  | (0.060) |  |  |  |  | (0.061) | (0.060) | (0.060) | (0.059) |  |  |  |  |  |  | (0.060) |
| Age: >66 years |  | 0.036 |  |  |  |  | 0.035 | 0.039 | 0.042 | 0.036 |  |  |  |  |  |  | 0.044 |
|  |  | (0.087) |  |  |  |  | (0.088) | (0.086) | (0.087) | (0.086) |  |  |  |  |  |  | (0.087) |
| Above Secondary |  | 0.064 |  |  |  |  | 0.061 | 0.068 | 0.066 | 0.065 |  |  |  |  |  |  | 0.069 |
|  |  | (0.065) |  |  |  |  | (0.061) | (0.065) | (0.062) | (0.065) |  |  |  |  |  |  | (0.060) |
| Marital Status |  |  |  |  |  |  |  |  |  |  |  |  |  |  |  |  |  |
| Single |  |  | 0.005 |  |  |  | -0.002 |  |  |  | 0.009 | 0.008 | 0.010 |  |  |  | 0.008 |
|  |  |  | (0.063) |  |  |  | (0.060) |  |  |  | (0.061) | (0.060) | (0.063) |  |  |  | (0.057) |
| Married |  |  | -0.023 |  |  |  | -0.023 |  |  |  | -0.022 | -0.023 | -0.019 |  |  |  | -0.017 |
|  |  |  | (0.020) |  |  |  | (0.020) |  |  |  | (0.019) | (0.021) | (0.020) |  |  |  | (0.020) |
| Job Status |  |  |  |  |  |  |  |  |  |  |  |  |  |  |  |  |  |
| Paid job |  |  |  | 0.188+ |  |  |  | 0.192+ |  |  | 0.190+ |  |  | 0.190+ | 0.198+ |  | 0.209+ |
|  |  |  |  | (0.113) |  |  |  | (0.114) |  |  | (0.114) |  |  | (0.113) | (0.118) |  | (0.120) |
| No-work paid |  |  |  | 0.162 |  |  |  | 0.151 |  |  | 0.154 |  |  | 0.157 | 0.172 |  | 0.150 |
|  |  |  |  | (0.131) |  |  |  | (0.130) |  |  | (0.129) |  |  | (0.130) | (0.134) |  | (0.129) |
| Unpaid job |  |  |  | 0.165+ |  |  |  | 0.167+ |  |  | 0.169+ |  |  | 0.165+ | 0.176+ |  | 0.183+ |
|  |  |  |  | (0.099) |  |  |  | (0.099) |  |  | (0.100) |  |  | (0.097) | (0.103) |  | (0.103) |
| ***Household Conditions*** |  |  |  |  |  |  |  |  |  |  |  |  |  |  |  |  |  |
| HAI:q2 |  |  |  |  | 0.013 |  |  |  | 0.014 |  |  | 0.013 |  | 0.013 |  | 0.014 | 0.015 |
|  |  |  |  |  | (0.085) |  |  |  | (0.085) |  |  | (0.085) |  | (0.086) |  | (0.085) | (0.085) |
| HAI:q3 |  |  |  |  | 0.078 |  |  |  | 0.080 |  |  | 0.079 |  | 0.080 |  | 0.081 | 0.086 |
|  |  |  |  |  | (0.094) |  |  |  | (0.097) |  |  | (0.094) |  | (0.095) |  | (0.094) | (0.098) |
| HAI:q4 |  |  |  |  | 0.051 |  |  |  | 0.055 |  |  | 0.053 |  | 0.052 |  | 0.058 | 0.065 |
|  |  |  |  |  | (0.089) |  |  |  | (0.091) |  |  | (0.090) |  | (0.090) |  | (0.090) | (0.093) |
| HE |  |  |  |  |  | -0.000 |  |  |  | -0.000 |  |  | -0.000 |  | -0.000 | -0.000 | -0.000 |
|  |  |  |  |  |  | (0.000) |  |  |  | (0.000) |  |  | (0.000) |  | (0.000) | (0.000) | (0.000) |
| 2018 | 0.245+ | 0.246+ | 0.245+ | 0.248+ | 0.245+ | 0.247+ | 0.245+ | 0.249+ | 0.245+ | 0.248+ | 0.248+ | 0.245+ | 0.247+ | 0.248+ | 0.250+ | 0.247+ | 0.251+ |
|  | (0.132) | (0.132) | (0.132) | (0.134) | (0.131) | (0.132) | (0.132) | (0.134) | (0.131) | (0.132) | (0.134) | (0.131) | (0.133) | (0.133) | (0.135) | (0.132) | (0.135) |
| Observations | 2,768 | 2,768 | 2,768 | 2,768 | 2,768 | 2,768 | 2,768 | 2,768 | 2,768 | 2,768 | 2,768 | 2,768 | 2,768 | 2,768 | 2,768 | 2,768 | 2,768 |

Notes: Bootstrapped standard errors in parenthesis (1,000 reps).+ p<0.1, * p<0.05, ** p<0.01, *** p<0.001. The first stage of Equations 1 and 3 are the same. Young adults, other marital status, does not know job status and, the first quartile of the household asset index (HAI) is used as reference categories. HE= monthly household expenditure in 2018 USD dollars. Coca production in hectares

Table A.7: DiD-IV second-stage results for TE equation

13

|  | M0 | M1 | M2 | M3 | M4 | M5 | M6 | M7 | M8 | M9 | M10 | M11 | M12 | M13 | M14 | M15 | M16 |
| --- | --- | --- | --- | --- | --- | --- | --- | --- | --- | --- | --- | --- | --- | --- | --- | --- | --- |
| ***Conflict-related violence*** |  |  |  |  |  |  |  |  |  |  |  |  |  |  |  |  |  |
| Conflict violence * time | -0.0481 | -0.0469 | -0.0495 | -0.0435 | -0.0463 | -0.0470 | -0.0478 | -0.0421 | -0.0453 | -0.0458 | -0.0452 | -0.0476 | -0.0482 | -0.0417 | -0.0424 | -0.0455 | -0.0406 |
|  | (0.2347) | (0.2211) | (0.2092) | (0.2090) | (0.2368) | (0.2385) | (0.2007) | (0.1941) | (0.2228) | (0.2242) | (0.1896) | (0.2120) | (0.2164) | (0.2106) | (0.2132) | (0.2406) | (0.1874) |
| 2018 | 0.2080* | 0.2028* | 0.2054* | 0.2032* | 0.2073* | 0.2052* | 0.2002* | 0.1977* | 0.2019* | 0.2000* | 0.2005* | 0.2045* | 0.2013* | 0.2024* | 0.2002* | 0.2049* | 0.1903* |
|  | (0.0918) | (0.0894) | (0.0841) | (0.0848) | (0.0927) | (0.0936) | (0.0830) | (0.0819) | (0.0901) | (0.0909) | (0.0789) | (0.0853) | (0.0871) | (0.0855) | (0.0867) | (0.0945) | (0.0808) |
| ***Age and Educ.*** |  |  |  |  |  |  |  |  |  |  |  |  |  |  |  |  |  |
| Age: 36-65 years |  | 0.0066 |  |  |  |  | 0.0007 | 0.0084 | 0.0091 | 0.0064 |  |  |  |  |  |  | 0.0037 |
|  |  | (0.0519) |  |  |  |  | (0.0514) | (0.0514) | (0.0522) | (0.0516) |  |  |  |  |  |  | (0.0517) |
| Age: 66 years |  | 0.0614 |  |  |  |  | 0.0542 | 0.0580 | 0.0671 | 0.0608 |  |  |  |  |  |  | 0.0556 |
|  |  | (0.0844) |  |  |  |  | (0.0852) | (0.0883) | (0.0836) | (0.0844) |  |  |  |  |  |  | (0.0890) |
| Above Secondary |  | -0.0348 |  |  |  |  | -0.0448 | -0.0433 | -0.0304 | -0.0349 |  |  |  |  |  |  | -0.0497 |
|  |  | (0.1126) |  |  |  |  | (0.1065) | (0.1132) | (0.1128) | (0.1129) |  |  |  |  |  |  | (0.1084) |
| Marital Status |  |  |  |  |  |  |  |  |  |  |  |  |  |  |  |  |  |
| Single |  |  | -0.0141 |  |  |  | -0.0142 |  |  |  | -0.0162 | -0.0154 | -0.0200 |  |  |  | -0.0231 |
|  |  |  | (0.0643) |  |  |  | (0.0629) |  |  |  | (0.0645) | (0.0647) | (0.0641) |  |  |  | (0.0626) |
| Married |  |  | -0.1035** |  |  |  | -0.1041** |  |  |  | -0.0997** | -0.1076** | -0.1108** |  |  |  | -0.1110** |
|  |  |  | (0.0374) |  |  |  | (0.0375) |  |  |  | (0.0367) | (0.0383) | (0.0369) |  |  |  | (0.0373) |
| Job Status |  |  |  |  |  |  |  |  |  |  |  |  |  |  |  |  |  |
| Paid job |  |  |  | -0.1511 |  |  |  | -0.1572 |  |  | -0.1418 |  |  | -0.1511 | -0.1664 |  | -0.1658 |
|  |  |  |  | (0.1927) |  |  |  | (0.1937) |  |  | (0.1935) |  |  | (0.1935) | (0.1863) |  | (0.1880) |
| No-work paid |  |  |  | 0.1078 |  |  |  | 0.0984 |  |  | 0.0819 |  |  | 0.1070 | 0.0937 |  | 0.0519 |
|  |  |  |  | (0.1794) |  |  |  | (0.1816) |  |  | (0.1814) |  |  | (0.1844) | (0.1731) |  | (0.1812) |
| Unpaid job |  |  |  | -0.1406 |  |  |  | -0.1461 |  |  | -0.1245 |  |  | -0.1413 | -0.1571 |  | -0.1487 |
|  |  |  |  | (0.1965) |  |  |  | (0.1962) |  |  | (0.1973) |  |  | (0.1976) | (0.1892) |  | (0.1899) |
| ***Household conditions*** |  |  |  |  |  |  |  |  |  |  |  |  |  |  |  |  |  |
| HAI: q2 |  |  |  |  | 0.0167 |  |  |  | 0.0168 |  |  | 0.0187 |  | 0.0152 |  | 0.0144 | 0.0140 |
|  |  |  |  |  | (0.0514) |  |  |  | (0.0512) |  |  | (0.0514) |  | (0.0520) |  | (0.0509) | (0.0507) |
| HAI: q3 |  |  |  |  | 0.0176 |  |  |  | 0.0181 |  |  | 0.0184 |  | 0.0140 |  | 0.0132 | 0.0092 |
|  |  |  |  |  | (0.0526) |  |  |  | (0.0522) |  |  | (0.0524) |  | (0.0520) |  | (0.0534) | (0.0518) |
| HAI: q4 |  |  |  |  | 0.0818 |  |  |  | 0.0828 |  |  | 0.0894 |  | 0.0792 |  | 0.0745 | 0.0769 |
|  |  |  |  |  | (0.0590) |  |  |  | (0.0601) |  |  | (0.0617) |  | (0.0576) |  | (0.0607) | (0.0623) |
| HE |  |  |  |  |  | 0.0001 |  |  |  | 0.0001 |  |  | 0.0001* |  | 0.0001 | 0.0001 | 0.0001+ |
|  |  |  |  |  |  | (0.0001) |  |  |  | (0.0001) |  |  | (0.0001) |  | (0.0001) | (0.0001) | (0.0001) |
| Observations | 2,618 | 2,618 | 2,618 | 2,618 | 2,618 | 2,618 | 2,618 | 2,618 | 2,618 | 2,618 | 2,618 | 2,618 | 2,618 | 2,618 | 2,618 | 2,618 | 2,618 |
| Kleibergen-Paap (KP) rk LM statistic+ | 3.82 | 3.84 | 3.82 | 3.82 | 3.83 | 3.84 | 3.84 | 3.84 | 3.84 | 3.85 | 3.82 | 3.83 | 3.84 | 3.83 | 3.84 | 3.84 | 3.86 |
| p-value of KP LM statistic | .0506 | .0502 | .0506 | .0506 | .0504 | .0502 | .0502 | .0502 | .05 | .0497 | .0506 | .0504 | .0502 | .0504 | .0501 | .05 | .0495 |
| Kleibergen-Paap Wald rk F++ | 22.9 | 23 | 22.9 | 22.5 | 22.9 | 23.1 | 23 | 22.5 | 23 | 23.2 | 22.4 | 22.9 | 23.1 | 22.5 | 22.7 | 23.1 | 22.6 |

Notes: Bootstrapped standard errors in parenthesis (1,000 reps).+ p<0.1, * p<0.05, ** p<0.01, *** p<0.001. Young adults, other marital status, does not know job status and, the first quartile of the household asset index (HAI) is used as reference categories. HE= monthly household expenditure in 2018 USD dollars. +Under identification test, ++Weak identification test.


Table A.8: DiD-IV first-stage results for NIE equation

14

|  | M0 | M1 | M2 | M3 | M4 | M5 | M6 | M7 | M8 | M9 | M10 | M11 | M12 | M13 | M14 | M15 | M16 |
| --- | --- | --- | --- | --- | --- | --- | --- | --- | --- | --- | --- | --- | --- | --- | --- | --- | --- |
| ***IV*** |  |  |  |  |  |  |  |  |  |  |  |  |  |  |  |  |  |
| Coca Production | -0.001*** | -0.001*** | -0.001*** | -0.001*** | -0.001*** | -0.001*** | -0.001*** | -0.001*** | -0.001*** | -0.001*** | -0.001*** | -0.001*** | -0.001*** | -0.001*** | -0.001*** | -0.001*** | -0.001*** |
|  | (0.000) | (0.000) | (0.000) | (0.000) | (0.000) | (0.000) | (0.000) | (0.000) | (0.000) | (0.000) | (0.000) | (0.000) | (0.000) | (0.000) | (0.000) | (0.000) | (0.000) |
| ***Mediator*** |  |  |  |  |  |  |  |  |  |  |  |  |  |  |  |  |  |
| AUDIT+ | 0.027 | 0.027 | 0.025 | 0.022 | 0.027 | 0.028 | 0.025 | 0.022 | 0.027 | 0.028 | 0.021 | 0.025 | 0.026 | 0.023 | 0.032 | 0.028 | 0.022 |
|  | (0.052) | (0.051) | (0.052) | (0.053) | (0.054) | (0.052) | (0.051) | (0.052) | (0.053) | (0.051) | (0.053) | (0.055) | (0.052) | (0.055) | (0.047) | (0.054) | (0.053) |
| ***Age and Educ.*** |  |  |  |  |  |  |  |  |  |  |  |  |  |  |  |  |  |
| Age: 36-65 years |  | -0.005 |  |  |  |  | -0.006 | -0.005 | -0.003 | -0.006 |  |  |  |  |  |  | -0.003 |
|  |  | (0.059) |  |  |  |  | (0.059) | (0.058) | (0.059) | (0.058) |  |  |  |  |  |  | (0.058) |
| Age: >66 years |  | 0.037 |  |  |  |  | 0.036 | 0.040 | 0.043 | 0.038 |  |  |  |  |  |  | 0.045 |
|  |  | (0.086) |  |  |  |  | (0.087) | (0.086) | (0.086) | (0.086) |  |  |  |  |  |  | (0.086) |
| Above Secondary |  | 0.065 |  |  |  |  | 0.062 | 0.069 | 0.067 | 0.066 |  |  |  |  |  |  | 0.070 |
|  |  | (0.066) |  |  |  |  | (0.063) | (0.066) | (0.063) | (0.066) |  |  |  |  |  |  | (0.060) |
| Marital Status |  |  |  |  |  |  |  |  |  |  |  |  |  |  |  |  |  |
| Single |  |  | 0.006 |  |  |  | -0.001 |  |  |  | 0.009 | 0.009 | 0.011 |  |  |  | 0.009 |
|  |  |  | (0.063) |  |  |  | (0.061) |  |  |  | (0.062) | (0.061) | (0.064) |  |  |  | (0.058) |
| Married |  |  | -0.022 |  |  |  | -0.021 |  |  |  | -0.020 | -0.022 | -0.018 |  |  |  | -0.016 |
|  |  |  | (0.022) |  |  |  | (0.022) |  |  |  | (0.020) | (0.024) | (0.022) |  |  |  | (0.021) |
| Job Status |  |  |  |  |  |  |  |  |  |  |  |  |  |  |  |  |  |
| Paid job |  |  |  | 0.182+ |  |  |  | 0.186+ |  |  | 0.185+ |  |  | 0.184+ | 0.105 |  | 0.203+ |
|  |  |  |  | (0.108) |  |  |  | (0.109) |  |  | (0.108) |  |  | (0.107) | (0.097) |  | (0.114) |
| No-work paid |  |  |  | 0.155 |  |  |  | 0.145 |  |  | 0.149 |  |  | 0.150 | -0.057 |  | 0.145 |
|  |  |  |  | (0.141) |  |  |  | (0.139) |  |  | (0.138) |  |  | (0.140) | (0.114) |  | (0.137) |
| Unpaid job |  |  |  | 0.160+ |  |  |  | 0.162+ |  |  | 0.164+ |  |  | 0.160+ | 0.089 |  | 0.178+ |
|  |  |  |  | (0.092) |  |  |  | (0.093) |  |  | (0.093) |  |  | (0.090) | (0.091) |  | (0.097) |
| ***Household Conditions*** |  |  |  |  |  |  |  |  |  |  |  |  |  |  |  |  |  |
| HAI: q2 |  |  |  |  | 0.012 |  |  |  | 0.014 |  |  | 0.013 |  | 0.012 |  | 0.013 | 0.015 |
|  |  |  |  |  | (0.085) |  |  |  | (0.085) |  |  | (0.085) |  | (0.086) |  | (0.085) | (0.085) |
| HAI: q3 |  |  |  |  | 0.078 |  |  |  | 0.080 |  |  | 0.079 |  | 0.080 |  | 0.081 | 0.086 |
|  |  |  |  |  | (0.094) |  |  |  | (0.097) |  |  | (0.094) |  | (0.095) |  | (0.094) | (0.098) |
| HAI: q4 |  |  |  |  | 0.049 |  |  |  | 0.053 |  |  | 0.051 |  | 0.050 |  | 0.056 | 0.064 |
|  |  |  |  |  | (0.089) |  |  |  | (0.091) |  |  | (0.090) |  | (0.090) |  | (0.090) | (0.093) |
| HE |  |  |  |  |  | -0.000 |  |  |  | -0.000 |  |  | -0.000 |  | -0.000 | -0.000 | -0.000 |
|  |  |  |  |  |  | (0.000) |  |  |  | (0.000) |  |  | (0.000) |  | (0.000) | (0.000) | (0.000) |
| 2018 | 0.246+ | 0.247+ | 0.246+ | 0.248+ | 0.246+ | 0.248+ | 0.246+ | 0.249+ | 0.246+ | 0.248+ | 0.248+ | 0.245+ | 0.248+ | 0.248+ | 0.277* | 0.248+ | 0.251+ |
|  | (0.132) | (0.132) | (0.133) | (0.134) | (0.131) | (0.133) | (0.132) | (0.134) | (0.131) | (0.133) | (0.135) | (0.132) | (0.133) | (0.134) | (0.128) | (0.132) | (0.135) |
| Observations | 2,768 | 2,768 | 2,768 | 2,768 | 2,768 | 2,768 | 2,768 | 2,768 | 2,768 | 2,768 | 2,768 | 2,768 | 2,768 | 2,768 | 2,618 | 2,768 | 2,768 |

Notes: Bootstrapped standard errors in parenthesis (1,000 reps).+ p<0.1, * p<0.05, ** p<0.01, *** p<0.001. Young adults, another marital status, does not know job status and, the first quartile of the household asset index (HAI) is used as reference categories. HE= monthly household expenditure in 2018 USD dollars. Coca production in hectares.


Table A.9: DiD-IV second-stage results for NIE equation

15

|  | M0 | M1 | M2 | M3 | M4 | M5 | M6 | M7 | M8 | M9 | M10 | M11 | M12 | M13 | M14 | M15 | M16 |
| --- | --- | --- | --- | --- | --- | --- | --- | --- | --- | --- | --- | --- | --- | --- | --- | --- | --- |
| ***Conflict-related violence*** |  |  |  |  |  |  |  |  |  |  |  |  |  |  |  |  |  |
| Conflict violence * time | -0.051 | -0.050 | -0.053 | -0.046 | -0.049 | -0.050 | -0.051 | -0.045 | -0.048 | -0.049 | -0.049 | -0.051 | -0.052 | -0.045 | -0.045 | -0.049 | -0.044 |
|  | (0.246) | (0.228) | (0.214) | (0.214) | (0.247) | (0.249) | (0.203) | (0.196) | (0.230) | (0.231) | (0.191) | (0.217) | (0.221) | (0.215) | (0.043) | (0.250) | (0.186) |
| ***Mediator*** |  |  |  |  |  |  |  |  |  |  |  |  |  |  |  |  |  |
| AUDIT+ | -0.052 | -0.052 | -0.061 | -0.050 | -0.055 | -0.053 | -0.062 | -0.051 | -0.055 | -0.053 | -0.059 | -0.065 | -0.063 | -0.054 | -0.052 | -0.055 | -0.065 |
|  | (0.042) | (0.042) | (0.040) | (0.041) | (0.043) | (0.042) | (0.041) | (0.041) | (0.043) | (0.042) | (0.039) | (0.041) | (0.040) | (0.042) | (0.034) | (0.043) | (0.041) |
| ***Age and Educ.*** |  |  |  |  |  |  |  |  |  |  |  |  |  |  |  |  |  |
| Age: 36-65 years |  | 0.004 |  |  |  |  | -0.002 | 0.006 | 0.007 | 0.004 |  |  |  |  |  |  | 0.001 |
|  |  | (0.052) |  |  |  |  | (0.051) | (0.051) | (0.052) | (0.051) |  |  |  |  |  |  | (0.051) |
| Age: 66 years |  | 0.059 |  |  |  |  | 0.052 | 0.056 | 0.065 | 0.059 |  |  |  |  |  |  | 0.053 |
|  |  | (0.085) |  |  |  |  | (0.086) | (0.089) | (0.084) | (0.085) |  |  |  |  |  |  | (0.089) |
| Above Secondary |  | -0.037 |  |  |  |  | -0.048 | -0.045 | -0.033 | -0.037 |  |  |  |  |  |  | -0.052 |
|  |  | (0.114) |  |  |  |  | (0.108) | (0.115) | (0.115) | (0.115) |  |  |  |  |  |  | (0.110) |
| Marital Status |  |  |  |  |  |  |  |  |  |  |  |  |  |  |  |  |  |
| Single |  |  | -0.016 |  |  |  | -0.016 |  |  |  | -0.017 | -0.018 | -0.022 |  |  |  | -0.024 |
|  |  |  | (0.064) |  |  |  | (0.063) |  |  |  | (0.065) | (0.065) | (0.064) |  |  |  | (0.063) |
| Married |  |  | -0.108** |  |  |  | -0.108** |  |  |  | -0.103** | -0.112** | -0.115** |  |  |  | -0.115** |
|  |  |  | (0.038) |  |  |  | (0.038) |  |  |  | (0.037) | (0.039) | (0.037) |  |  |  | (0.038) |
| Job Status |  |  |  |  |  |  |  |  |  |  |  |  |  |  |  |  |  |
| Paid job |  |  |  | -0.145 |  |  |  | -0.152 |  |  | -0.135 |  |  | -0.145 | -0.161 |  | -0.159 |
|  |  |  |  | (0.187) |  |  |  | (0.188) |  |  | (0.188) |  |  | (0.188) | (0.148) |  | (0.182) |
| No-work paid |  |  |  | 0.112 |  |  |  | 0.102 |  |  | 0.086 |  |  | 0.111 | 0.098 |  | 0.055 |
|  |  |  |  | (0.175) |  |  |  | (0.177) |  |  | (0.177) |  |  | (0.180) | (0.156) |  | (0.177) |
| Unpaid job |  |  |  | -0.139 |  |  |  | -0.145 |  |  | -0.122 |  |  | -0.140 | -0.156 |  | -0.147 |
|  |  |  |  | (0.193) |  |  |  | (0.192) |  |  | (0.193) |  |  | (0.194) | (0.167) |  | (0.185) |
| ***Household Conditions*** |  |  |  |  |  |  |  |  |  |  |  |  |  |  |  |  |  |
| HAI: q2 |  |  |  |  | 0.019 |  |  |  | 0.019 |  |  | 0.021 |  | 0.017 |  | 0.016 | 0.017 |
|  |  |  |  |  | (0.052) |  |  |  | (0.052) |  |  | (0.052) |  | (0.053) |  | (0.052) | (0.052) |
| HAI: q3 |  |  |  |  | 0.020 |  |  |  | 0.020 |  |  | 0.021 |  | 0.016 |  | 0.015 | 0.011 |
|  |  |  |  |  | (0.054) |  |  |  | (0.053) |  |  | (0.053) |  | (0.053) |  | (0.054) | (0.053) |
| HAI: q4 |  |  |  |  | 0.085 |  |  |  | 0.086 |  |  | 0.094 |  | 0.082 |  | 0.078 | 0.081 |
|  |  |  |  |  | (0.060) |  |  |  | (0.061) |  |  | (0.063) |  | (0.059) |  | (0.062) | (0.064) |
| HE |  |  |  |  |  | 0.000 |  |  |  | 0.000 |  |  | 0.000* |  | 0.000+ | 0.000 | 0.000+ |
|  |  |  |  |  |  | (0.000) |  |  |  | (0.000) |  |  | (0.000) |  | (0.000) | (0.000) | (0.000) |
| 2018 | 0.207* | 0.202* | 0.204* | 0.203* | 0.207* | 0.204* | 0.199* | 0.197* | 0.201* | 0.199* | 0.200* | 0.203* | 0.200* | 0.202* | 0.200*** | 0.204* | 0.190* |
|  | (0.096) | (0.092) | (0.086) | (0.087) | (0.097) | (0.098) | (0.084) | (0.083) | (0.093) | (0.094) | (0.080) | (0.088) | (0.089) | (0.088) | (0.023) | (0.098) | (0.081) |
| Observations | 2,618 | 2,618 | 2,618 | 2,618 | 2,618 | 2,618 | 2,618 | 2,618 | 2,618 | 2,618 | 2,618 | 2,618 | 2,618 | 2,618 | 2,618 | 2,618 | 2,618 |
| Kleibergen-Paap (KP) rk LM statistic+ | 3.85 | 3.86 | 3.85 | 3.85 | 3.85 | 3.86 | 3.86 | 3.86 | 3.87 | 3.87 | 3.85 | 3.85 | 3.86 | 3.85 | 3.86 | 3.87 | 3.88 |
| p-value of KP LM statistic | .0499 | .0495 | .0499 | .0498 | .0497 | .0494 | .0495 | .0495 | .0493 | .0491 | .0498 | .0497 | .0495 | .0496 | .0494 | .0493 | .0489 |
| Kleibergen-Paap Wald rk F++ | 23.3 | 23.3 | 23.2 | 22.8 | 23.3 | 23.4 | 23.3 | 22.8 | 23.3 | 23.5 | 22.7 | 23.2 | 23.4 | 22.8 | 23 | 23.4 | 22.9 |

Notes: Bootstrapped standard errors in parenthesis (1,000 reps).+ p<0.1, * p<0.05, ** p<0.01, *** p<0.001. Young adults, another marital status, does not know job status and, the first quartile of the household asset index (HAI) is used as reference categories. HE= monthly household expenditure in 2018 USD dollars. +Under identification test, ++Weak identification test.


Table A.10: DiD-IV second-stage results for NDE equation

16

|  | M0 | M1 | M2 | M3 | M4 | M5 | M6 | M7 | M8 | M9 | M10 | M11 | M12 | M13 | M14 | M15 | M16 |
| --- | --- | --- | --- | --- | --- | --- | --- | --- | --- | --- | --- | --- | --- | --- | --- | --- | --- |
| ***Conflict-related violence*** |  |  |  |  |  |  |  |  |  |  |  |  |  |  |  |  |  |
| Conflict violence * time | -0.056 | -0.053 | -0.057 | -0.059 | -0.055 | -0.056 | -0.054 | -0.056 | -0.052 | -0.053 | -0.060 | -0.056 | -0.056 | -0.058 | -0.058 | -0.055 | -0.056 |
|  | (0.301) | (0.284) | (0.290) | (0.276) | (0.288) | (0.279) | (0.277) | (0.258) | (0.271) | (0.263) | (0.267) | (0.278) | (0.270) | (0.264) | (0.256) | (0.268) | (0.227) |
| 2018 | -0.012 | -0.012 | -0.016 | -0.006 | -0.013 | -0.013 | -0.015 | -0.005 | -0.013 | -0.013 | -0.008 | -0.016 | -0.017 | -0.006 | -0.006 | -0.013 | -0.009 |
|  | (0.111) | (0.107) | (0.109) | (0.104) | (0.107) | (0.104) | (0.106) | (0.099) | (0.103) | (0.100) | (0.102) | (0.105) | (0.102) | (0.100) | (0.097) | (0.100) | (0.090) |
| ***Age and Educ.*** |  |  |  |  |  |  |  |  |  |  |  |  |  |  |  |  |  |
| Age: 36-65 years |  | -0.046 |  |  |  |  | -0.049 | -0.043 | -0.045 | -0.046 |  |  |  |  |  |  | -0.046 |
|  |  | (0.049) |  |  |  |  | (0.048) | (0.048) | (0.048) | (0.049) |  |  |  |  |  |  | (0.045) |
| Age: 66 years |  | -0.037 |  |  |  |  | -0.040 | -0.031 | -0.035 | -0.037 |  |  |  |  |  |  | -0.032 |
|  |  | (0.062) |  |  |  |  | (0.060) | (0.061) | (0.059) | (0.061) |  |  |  |  |  |  | (0.057) |
| Above Secondary |  | -0.049 |  |  |  |  | -0.053 | -0.033 | -0.047 | -0.049 |  |  |  |  |  |  | -0.036 |
|  |  | (0.074) |  |  |  |  | (0.072) | (0.074) | (0.075) | (0.072) |  |  |  |  |  |  | (0.072) |
| Marital Status |  |  |  |  |  |  |  |  |  |  |  |  |  |  |  |  |  |
| Single |  |  | -0.035 |  |  |  | -0.031 |  |  |  | -0.020 | -0.035 | -0.038 |  |  |  | -0.020 |
|  |  |  | (0.070) |  |  |  | (0.066) |  |  |  | (0.068) | (0.068) | (0.070) |  |  |  | (0.064) |
| Married |  |  | -0.068* |  |  |  | -0.069* |  |  |  | -0.060+ | -0.070* | -0.071* |  |  |  | -0.065+ |
|  |  |  | (0.035) |  |  |  | (0.034) |  |  |  | (0.034) | (0.035) | (0.036) |  |  |  | (0.035) |
| Job Status |  |  |  |  |  |  |  |  |  |  |  |  |  |  |  |  |  |
| Paid job |  |  |  | 0.112 |  |  |  | 0.108 |  |  | 0.116 |  |  | 0.112 | 0.107 |  | 0.108 |
|  |  |  |  | (0.122) |  |  |  | (0.119) |  |  | (0.124) |  |  | (0.122) | (0.127) |  | (0.127) |
| No-work paid |  |  |  | 0.081 |  |  |  | 0.077 |  |  | 0.065 |  |  | 0.079 | 0.077 |  | 0.051 |
|  |  |  |  | (0.148) |  |  |  | (0.148) |  |  | (0.151) |  |  | (0.152) | (0.153) |  | (0.162) |
| Unpaid job |  |  |  | 0.029 |  |  |  | 0.027 |  |  | 0.037 |  |  | 0.028 | 0.024 |  | 0.030 |
|  |  |  |  | (0.125) |  |  |  | (0.122) |  |  | (0.126) |  |  | (0.125) | (0.130) |  | (0.130) |
| ***Household Conditions*** |  |  |  |  |  |  |  |  |  |  |  |  |  |  |  |  |  |
| HAI: q2 |  |  |  |  | 0.038 |  |  |  | 0.038 |  |  | 0.039 |  | 0.039 |  | 0.037 | 0.039 |
|  |  |  |  |  | (0.026) |  |  |  | (0.026) |  |  | (0.025) |  | (0.025) |  | (0.026) | (0.025) |
| HAI: q3 |  |  |  |  | 0.037 |  |  |  | 0.035 |  |  | 0.037 |  | 0.038 |  | 0.036 | 0.035 |
|  |  |  |  |  | (0.029) |  |  |  | (0.029) |  |  | (0.030) |  | (0.029) |  | (0.031) | (0.031) |
| HAI: q4 |  |  |  |  | 0.060 |  |  |  | 0.058 |  |  | 0.065 |  | 0.062 |  | 0.059 | 0.061 |
|  |  |  |  |  | (0.044) |  |  |  | (0.043) |  |  | (0.042) |  | (0.041) |  | (0.045) | (0.040) |
| HE |  |  |  |  |  | 0.000 |  |  |  | 0.000 |  |  | 0.000 |  | 0.000 | 0.000 | 0.000 |
|  |  |  |  |  |  | (0.000) |  |  |  | (0.000) |  |  | (0.000) |  | (0.000) | (0.000) | (0.000) |
| Observations | 2,618 | 2,618 | 2,618 | 2,618 | 2,618 | 2,618 | 2,618 | 2,618 | 2,618 | 2,618 | 2,618 | 2,618 | 2,618 | 2,618 | 2,618 | 2,618 | 2,618 |
| Kleibergen-Paap (KP) rk LM statistic+ | 3.82 | 3.84 | 3.82 | 3.82 | 3.83 | 3.84 | 3.84 | 3.84 | 3.84 | 3.85 | 3.82 | 3.83 | 3.84 | 3.83 | 3.84 | 3.84 | 3.86 |
| p-value of KP LM statistic | .0506 | .0502 | .0506 | .0506 | .0504 | .0502 | .0502 | .0502 | .05 | .0497 | .0506 | .0504 | .0502 | .0504 | .0501 | .05 | .0495 |
| Kleibergen-Paap Wald rk F++ | 22.9 | 23 | 22.9 | 22.5 | 22.9 | 23.1 | 23 | 22.5 | 23 | 23.2 | 22.4 | 22.9 | 23.1 | 22.5 | 22.7 | 23.1 | 22.6 |

Notes: Bootstrapped standard errors in parenthesis (1,000 reps).+ p<0.1, * p<0.05, ** p<0.01, *** p<0.001. Dependent variable: AUDIT. Young adults, another marital status, does not know job status and, the first quartile of the household asset index (HAI) is used as reference categories. HE= monthly household expenditure in 2018 USD dollars. +Under identification test, ++Weak identification test.


**REFERENCES**

Huber, M. (2019). A review of causal mediation analysis for assessing direct and indirect treatment effects. In *FSES Working Papers* (500; FSES Working Papers). Faculty of Economics and Social Sciences, University of Freiburg/Fribourg Switzerland. https://ideas.repec.org/p/fri/fribow/fribow00500.html

1. Although, there are several parameters of interest in causal mediation analysis, for example, refer to Huber (2019) for further details, in this study we estimate the natural direct and indirect effects (Huber, 2019). [↑](#footnote-ref-1)
